# Supplementary material for: Pangenome-based genome inference allows efficient and accurate genotyping across a wide spectrum of variant classes
Source: Nat Genet. 2022 Apr 11;54(4):518–25. doi: 10.1038/s41588-022-01043-w (PMC9005351; doi:10.1038/s41588-022-01043-w)
Supplement: Supplementary file 1 — Supplementary Sections 1–7, Figs. 1–20, Tables 1–5 and 7–8. [file 41588_2022_1043_MOESM1_ESM.pdf]

nature portfolio

https://doi.org/10.1038/s41588-022-01043-w

Supplementary information

Pangenome-based genome inference allows efficient and accurate genotyping across a wide spectrum of variant classes

In the format provided by the authors and unedited

Pangenome-based Genome Inference - Supplementary Material

Jana Ebler[1], Peter Ebert[1], Wayne E. Clarke[2], Tobias Rausch[3,4], Peter A. Audano[5], Torsten Houwaart[7], Yafei Mao[5], Jan Korbel[3], Evan E. Eichler[5,6], Michael C. Zody[2], Alexander T. Dilthey[7,8,9], and Tobias Marschall[1,*]

[1]Institute for Medical Biometry and Bioinformatics, Medical Faculty, Heinrich Heine University Düsseldorf, Düsseldorf, Germany
[2]New York Genome Center, New York, New York, USA
[3]European Molecular Biology Laboratory (EMBL), Genome Biology Unit, Heidelberg, Germany
[4]European Molecular Biology Laboratory (EMBL), GeneCore, Heidelberg, Germany
[5]Department of Genome Sciences, University of Washington School of Medicine, Seattle, Washington, USA
[6]Howard Hughes Medical Institute, University of Washington, Seattle, Washington, USA
[7]Institute of Medical Microbiology and Hospital Hygiene, Heinrich Heine University Düsseldorf, Düsseldorf, Germany
[8]Institute of Medical Statistics and Computational Biology, University of Cologne, Cologne, Germany
[9]Cologne Excellence Cluster on Cellular Stress Responses in Aging-Associated Diseases (CECAD), University of Cologne, Cologne, Germany

1 Variant Calling and Pangenome reference construction

The input to our genotyping algorithm is a reference genome (FASTA-file), short-read sequencing reads (FASTQ format) and a multisample VCF file that defines a pangenome graph containing variants and known haplotype sequences. In order to create such an input graph, we have developed a pipeline which calls variants from haplotype resolved assemblies as described below and uses them to construct a pangenome representation. However, we want to stress that our tool is not restricted to VCFs created in this way and in fact can be run with any fully phased, multisample VCF file.

1.1 Callset statistics

Supplementary Table 2 shows the number of variants at different stages of our variant calling pipeline. We call variants on each individual haplotype (first column). The second column shows the number of variants left after removing regions covered in less than 20% of the samples. The third column corresponds to the final callset and lists the number of variants left after filtering out positions with an mendelian error in a least one of the trios. In the last column, we show the number of bubbles in the pangenome resulting from inserting all callset variants into the linear reference. Our variant calling pipeline calls SNPs, insertions and deletions. We distinguish small (1-19bp), midsize (20-49bp) and large ($\geq$ 50bp) variants. When constructing the pangenome graph, variation will be represented by bubbles in the graph. Sets of overlapping variant alleles will be combined into muli-allelic bubbles (i.e. bubbles with more than two branches). We therefore distinguish simple, "biallelic" bubbles that consist of two branches and can be easily classified as SNP, insertion or deletion, and "complex" bubbles with more than two branches representing more complex variation (Extended Data Figure 1). We counted the number of branches of each bubble in the graph and plotted it as a function of its reference length (Supplementary Figure 1). While the number of branches is below 5 for the majority of bubbles, it tends to be higher especially for larger bubbles representing more complex regions of high variability.

Supplementary Table 3 shows sample-specific variant numbers for all samples that were used for variant calling. For each sample, we show the total number of variants present in at least one of its haplotypes, i.e. all variants for which the sample has a genotype different from 0/0 (total). Additionally, we show the number of variants unique to the sample (unique), i.e. variants not seen in any of the other samples. All variants that are unique to a sample will not be genotypable by any re-genotyping approach and we later exclude these variants for evaluation.

2 Comparison to existing approaches

2.1 Evaluation metrics

2.1.1 Weighted genotype concordance

Each genotyped variant is either absent from the truth set (0/0, in case it is not present in the left out sample), heterozygous (0/1) or homozygous (1/1). We construct a confusion matrix counting all cases (Supplementary Figure 2). The counts on

the diagonal (labelled T_0/0, T_0/1, T_1/1) correspond to correctly genotyped variants. All others are errors. For all three genotypes, we compute the concordances by counting the number of correct predictions and divide it by the total number of variants in that category:

$$\text{conc}(0/0) = \frac{\text{T\_0/0}}{\text{T\_0/0} + \text{F\_0/0}} \qquad \text{conc}(0/1) = \frac{\text{T\_0/1}}{\text{T\_0/1} + \text{F\_0/1}} \qquad \text{conc}(1/1) = \frac{\text{T\_1/1}}{\text{T\_1/1} + \text{F\_1/1}}$$

Since we genotype all variants detected across multiple samples (including many rare alleles) in our evaluation sample, the majority of variants will be absent in the evaluation sample. That is, the number of variants whose true genotype is 0/0, will be higher compared to the ones with genotypes 0/1 or 1/1. To adjust for unqeual numbers of 0/0, 0/1 and 1/1 genotypes in our ground truth, we compute the weighted genotype concordance as:

$$\text{weighted genotype concordance} = \frac{\text{conc}(0/0) + \text{conc}(0/1) + \text{conc}(1/1)}{3}$$

As mentioned previously, we exclude all variants unique to the evaluation sample when computing the weighted genotype concordance, since these variants are not part of the set of input variants given to all genotypers and thus will not be considered for genotyping (as all tools re-genotype variants and do not detect them).

2.1.2 Fraction of genotyped variants

Many of the re-genotyping tools we consider can report genotypes "./." for input variants that they are not able to genotype. For each tool, we compute the fraction of input variants that were reported with such an "untyped" genotype.

2.1.3 (Adjusted) Precision/Recall/F-score

We use `RTG vcfeval`[1] in order to compute precision and recall for our genotype predictions. We compute two versions of precision, recall and F-scores: taking all variants into account (including those that are unique to the evaluation sample and hence missing from the input set and undetectable by regenotyping, see Supplementary Table S3), and an *adjusted* version, where we remove all variants unique to the evaluation sample from the truth set. Therefore, the unadjusted version combines the effects of variants missing the input set to be genotyped and the performance of the genotyping method, while the adjusted version aims to only measure the performance of the method (and does not penalize variants absent in the input set). True positives, false positives and false negatives are defined as shown in Supplementary Figure 2[1] and precision, recall and F-score are defined as:

$$\text{precision} = \frac{TP}{TP + FP} \qquad \text{recall} = \frac{TP}{TP + FN} \qquad \text{F-score} = 2 \cdot \frac{\text{precision} \cdot \text{recall}}{\text{precision} + \text{recall}}$$

2.1.4 Note on Precision/Recall/F-score metrics

We offer precision/recall/F-score metrics to facilitate comparison to other studies, including on methods for variant calling. However, these metrics come with the following caveats when evaluating a re-genotyping experiments and should hence be interpreted accordingly: The more samples we use to generate the set of known variants to genotype in a new sample, the larger the amount of rare variants and thus the larger the fraction of variants whose true genotype of the new sample is 0/0. That is, we can make our set of input variants (almost) arbitrarily large by adding variants absent in the new sample. The possibility of adding noise when including a large number of rare alleles when constructing pangenome representations is a known effect and an important consideration[2]. As a consequence, the number of false positive calls will increase with the increase of the number of tested variants, while the number of true positive calls is limited by the actual number of variants present in the new sample, reducing the precision. An example is shown in Supplementary Figure 3a. This also explains why the precision we see for all genotypers in our evaluation is sometimes small compared to the genotype concordance (Supplementary Figure 3b).

2.2 Leave-one-out Experiment

2.2.1 Using assembly-based calls as ground truth

We ran the "leave-out-one" experiment (Methods, Extended Data Figure 2) for samples NA12878 and NA24385. We computed the weighted genotype concordance, adjusted precision and recall, and the adjusted F-score for evaluation when comparing to our ground truth set containing all variants detected in the haplotype-resolved assemblies of the left out sample. Results for the different evaluation regions (Methods) are shown in Supplementary Figures 4-9. In addition to running all tools in re-genotyping mode on our provided variants, we ran GATK and Platypus in discovery mode and let them detect their own variant set. We evaluated the results based on the same truth set computing adjusted precision, recall and F-scores to enable a

direct comparison of re-genotyping and discovery. Results are shown in Supplementary Figure 10. Results show that especially Platypus benefits from re-typing assembly-based calls, as it struggles making accurate calls in STR/VNTR regions and complex regions of the genome that are usually poorly accessible by short read alignments.

2.2.2 Using GIAB small variants as ground truth

We used the GIAB small variant benchmark[3] as another ground truth set in order to evaluate our "leave-one-out" results and computed the adjusted precision and recall for evaluation by removing all variants absent from our callsets for evaluation. In addition to re-genotyping our variant callset, we again ran GATK and Platypus in discovery mode for comparison and computed precision and recall in addition to their adjusted versions. Results are shown in Supplementary Figure 11. Using the discovery mode for GATK and Platypus, results for the adjusted and non-adjusted versions are very similar, while for the re-typing tools, the recall drops when using the un-adjusted metric. This is expected, since the adjustment would ignore variants absent from the input panel. These positions are not present in our callset, as they have not been seen in the samples used for variant calling and thus, are also not considered for genotyping. As a consequence these variants will be all counted as false negatives, reducing the recall. This causes the curves for all re-typing tools to shift to the left. The discovery tools are not effected by this, since they are able to discover variants themselves.

2.2.3 Using syndip SVs as ground truth

We used the SVs ($\geq$ 50 bp) contained in the syndip benchmark set[4] as an additional ground truth set for evaluating our genotyping. As the syndip sample is not part of our assembly samples, we used our callset created from all eleven assembly samples as input for genotyping. As before, we computed the weighted genotype concordance and the adjusted precision and recall metrics for evaluation. In order to define the set of *untypable* variants, we ran `RTG vcfeval`[1] in order to detect all syndip variants that are absent from our callset and excluded them when computing our evaluation metrics (as they cannot be genotyped by any re-genotyping approach). Results are shown in Supplementary Figure 12.

2.3 Resources.

2.3.1 Comparison of runtimes and memory usages

The runtime and peak memory usage of all genotypers is presented in in Supplementary Table 5. For all methods, we measured the resources needed to produce genotypes given the raw, unaligned sequencing reads (“total”) as well as the resources needed specifically for genotyping (“genotyping”). For the mapping-based approaches (Platypus, GATK, Paragraph, GraphTyper and Giraffe) the latter excludes the resources needed for aligning the sequencing reads, for the k-mer based approaches (PanGenie and BayesTyper) it excludes the resources needed for counting k-mers. Note that not all tools are able to genotype all considered variant types. We ran GATK only on SNPs, small and midsize variants. Paragraph was only run on midsize and large variants and GraphTyper only on large variants. We ran Giraffe only for sample NA12878 at coverage 30$\times$ and only on large variants, as we observed a very high runtime for its graph alignment step. All tools were run on a HPC-cluster predominantly consisting of Intel E5-2697v2 (2$\times$ 12 cores and 128 GB of RAM) and Intel Xeon Gold 6136 (2$\times$ 12 cores and 192 GB of RAM) nodes.

2.3.2 Asymptotic runtime of PanGenie

PanGenie is based on a Hidden Markov Model which, for each variant position, defines one state for each pair of haplotypes of the input panel. Given n variants to be genotyped and m panel haplotypes (which equals twice the number of samples), there will be $O(m^2 \cdot n)$ states. Applying the Forward-Backward algorithm to the HMM thus corresponds to a runtime quadratic in the number of states. If the number of panel haplotypes grows, the algorithm will get slow. To tackle this problem, we have implemented a subsampling step, which repeatedly subsamples sets of haplotypes from the full panel and genotypes all variants in each subset. Genotype predictions resulting from each of these subsets are later combined to obtain the final genotype likelihoods. Assume we split the set of m input haplotypes in l subsets each of a fixed size k. PanGenie's genotyping step is now run separately on each of the l sets in time $O(k^4 \cdot n)$. This will result in a total runtime linear in the number of subsets, i.e. $O(l \cdot k^4 \cdot n)$. PanGenie automatically switches to this subsampling mode if the number of input haplotypes exceeds 30. For all experiments in this paper, we ran PanGenie without subsampling using the full HMM.

3 Genotyping HLA genes

To evaluate the accuracy of all 14 haplotype-resolved assemblies in the HLA region, we used HLA*ASM[5] to determine assembly HLA types (Supplementary Table 6). HLA*ASM successfully processed 27 out of 28 input assemblies and identified perfect (edit distance 0) HLA G group matches[6] for all classical HLA loci (HLA-A, -B, -C, -DQA1 -DQB1, -DRB1) in all processed input assemblies with one exception (HLA-DRB1 in NA19238), which was resolved by manual curation[7]. To verify the accuracy of the assembly HLA types, we integrated publicly available HLA genotype data for 1000 Genomes samples[8–10] for HLA-A, -B, -C, -DQB1, and -DRB1, intersected these with the assembly-implied HLA types, and found perfect agreement in all evaluated cases (9 samples and 85 individual genotype comparisons, Supplementary Table 6.

We analyzed our genotyping performance inside of the MHC region. Since we used a reference genome containing alternative HLA contigs, the MHC region was not covered well by our callset described previously (Section *Constructing a pangenome reference*). We therefore used the same pipeline to generate a second version of our variant calls and pangenome graph using a reference genome that contains only chromosomes 1-22, chromosome X and chromosome Y. We analyzed the MHC region by repeating the leave-one-out experiment described earlier with this new callset and evaluated genotyping performance for commonly studied HLA-genes. We ran our leave-one-out experiment for three samples: HG00731, NA12878 and NA24385. Analogously to what we described in Methods and Extended Data Figure 2, we repeatedly construct callsets and pangenome graphs excluding the respective samples and evaluate genotypes by comparing to the variants detected in the left out sample. As mentioned previously, we restrict our evaluation to all variants that are genotypable and exclude such that are unique to the left out sample.

We present weighted genotype concordances that we obtained for the HLA genes in Extended Data Figure 9. We separately evaluate variants (all types) located in biallelic regions of the genome and such located in regions with complex bubbles in the pangenome graph. Our callset did not fully cover the C4 genes (C4A and C4B) since the region was not completely covered by contig alignments in most haplotypes (including one of the haplotypes of NA12878) possibly due to the presence of large structural variants in this region. Thus, the evaluation for these genes only corresponds to the parts that were accessible for variant calling (Supplementary Figure 13).

4 Genotyping Larger Cohorts

We randomly selected 100 trios (20 of each superpopulation: AFR,AMR,EAS,EUR,SAS) that are part of the 1000 Genomes Project and genotyped all our variant calls across these 300 samples. We used the pangenome graph containing all eleven assembly samples as an input for PanGenie. Our callset might contain variants that are difficult to genotype correctly. Our goal is to identify a high quality subset of variants that we can reliably genotype. For this purpose, we define different filters based on the predicted genotypes that we will list below. One metric used for defining filters is the mendelian consistency. We computed the mendelian consistency for each variant by counting the number of trios for which the predicted genotypes are consistent with Mendelian laws. We only consider trios with at least two different genotypes, that is, we exclude a trio if all three genotypes are 0/0, 0/1 or 1/1. This results in a more strict definition of mendelian consistency. For the unfiltered variant set, the mean mendelian consistency for SNPs was 0.98, for small variants between 0.93-0.95, for midsize variants between 0.90-0.93 and for large variants we observed numbers between 0.88-0.89 (Supplementary Figure 14). In addition to genotyping all 300 trio samples we also genotyped all eleven panel samples using the full input panel. Genotyping samples that are also in the input graph can help us to find cases where panel haplotypes and reads disagree and thus is another useful filter criterion. We define filters as follows:

- **ac0-fail**: a variant fails this filter, if it was genotyped with allele frequency 0.0 across all samples.
- **mendel-fail**: a variant fails this filter if the fraction of mendelian consistent trios was below 90%. Our definition of mendelian consistency excludes all trios with all 0/0, all 0/1 or all 1/1 genotypes and only considers such with at least two different genotypes.
- **gq-fail**: a variant fails this filter if it was genotyped with a genotype quality below 200 in less than 5 samples.
- **self-fail**: in addition to the 100 trios, we also genotyped the 11 panel samples. A variant fails this filter, if the genotype concordance across all panel samples was below 90%.
- **non-ref-fail**: the variant was genotyped as 0/0 across all panel samples.

For all combinations of filters, we show the number of large deletions and large insertions in each category in Supplementary Figure 15. In order to define a strict, high quality set of variants, we select those that passed all five filters. This rather stringent set of variants contains about 93% of all SNPs, between 62-67% of all small insertions and deletions, and about 50% of all midsize and large variants (Supplementary Table 7). For quality control, we analyzed allele frequencies and the fraction of heterozygous genotypes for all variants contained in our unfiltered and strict sets (Supplementary Figures 16 and 17). Additionally, we used VCFTools[11] to test the genotype predictions of all variants typed with an allele frequency > 0.0 for conformance with Hardy-Weinberg equilibrium and corrected for multiple hypothesis testing by applying Benjamini-Hochberg correction[12] ($\alpha = 0.05$). For both sets, the majority of variants behave as expected by Hardy-Weinberg equilibrium. For the unfiltered set, between 87-91% of all variants/types show no significant deviation from HWE in non-repetitive regions. In repetitive regions, the fractions are between 83-90%. For the strict set, we observed numbers between $88-93\%$ in non-repetitive regions and $89-94\%$ in repeat regions (Supplementary Figures 16, 17).

In addition to defining a strict set, we constructed a more lenient set for our SV calls ($\geq$ 50bp) using a machine learning approach based on support vector regression. We use the strict set as positive set and define a negative set consisting of all variants that were typed with an allele frequency $>$ 0.0 and failed at least 3 filters. For large insertions, the negative set contained 2,611 variants, for large deletions 1,125. The model then predicts scores between -1 (worst) and 1 (best) for all variants that are neither in the positive nor the negative set. We show the distribution of scores for our variant calls in Supplementary Figure 18a . The lenient set is then constructed by adding all variants with a score above -0.5 to our strict SV set. We show the number of variants contained in the strict and lenient sets in Supplementary Table 8. For large insertions and deletions, the lenient set contains around 78% and 83% of all variants, respectively, while showing statistics similar to the strict set (Supplementary Figure 18).

We compared our variant calls to the Genome in a Bottle set of medically relevant SVs[13]. Our unfiltered callset contained 209 of all 250 medically relevant SVs. We further analyzed which fraction of these made it into our strict and lenient sets. We observed that 174 medically relevant SVs were contained in our lenient set, of which 119 were part of our strictly filtered set. We show the score distribution for these variants as well as allele frequencies and heterozygosities observed across all 200 unrelated samples for the lenient set in Extended Data Figure 10.

5 Comparison to gnomAD

We compared the variant calls that we obtained from haplotype-resolved assemblies of eleven individuals to the variants that are part of the Genome Aggregation Database (gnomAD)[14]. gnomAD contains 433,371 structural variants collected across 14,891 genomes from different populations. Since gnomaD calls were generated relative to reference genome version GRCh19, we used UCSC liftOver (`https://genome.ucsc.edu/cgi-bin/hgLiftOver`) to convert their coordinates to GRCh38. For comparison, we used all our SV calls that were contained in our lenient set. We define two variant calls to be the same if their reciprocal overlap is at least 50% or their start, end and allele lengths deviate by less than 200bp. Based on these criteria, we found that both callsets had 34,468 variants in common. 344,815 variants were only contained in gnomAD and 84,658 were only in our assembly callset. 51% of the 34,468 variants in the intersection are located inside of STR/VNTR regions. For the variants contained only in our assembly callset, this percentage is around 80%.

6 LD analysis

We performed a linkage disequilibrium (LD) analysis based on the genotypes we obtained across all 200 unrelated samples. We used `gatk4`[15] to annotate the calls with variant ids from dbSNP[16]. We selected variants that are contained in the NHGRI-EBU GWAS catalog[17] and used `plink`[18] to determine structural variants that are in LD with the GWAS variants ($r^2 \geq 0.8$). For 147 disease-associated GWAS variants we found a SV in linkage disequilibrium. We list all SVs in strong LD with GWAS variants ($r^2 \geq 0.9$) in Supplementary Table 9.

Our linkage disequilibrium analysis showed one interesting insertion of length 129 bp located at position 133,278,856 of chromosome 9, that was in LD with six GWAS SNPs (rs2519093, rs495828, rs507666, rs579459, rs635634 and rs651007). Supplementary Figure 19 shows dot pairwise dot plots of the insertion sequence, LTR10B2 consensus sequence and the reference sequence of this region (LTR-annotated sequence from GRCh38). It indicates that the insertion sequence contains 3 exact copies of “TAACGCAGTTTCTGTTTCTGTGTCCTTCCCCTATTGGCTGGGG" (43bp) and suggests that this sequence is expanded from one to four copies.

The other interesting case was a 322 bp insertion inside of the CCDC91 gene at position 28,264,365 of chromosome 12 (Supplementary Figure 20). It was in LD with two GWAS variants (rs10843151 and rs11049566) both linked to body fat[17] and is located close to regulatory element E1601673/enhD reported by ENCODE[19].

7 Command lines used for variant calling and genotyping

7.1 Assembly-based variant calling and pangenome graph construction

For variant calling, contigs of each haplotype were aligned against the reference genome using minimap2 (version 2.18)

```
minimap2 -cx asm20 -m 10000 -z 10000,50 -r 50000 --end-bonus=100 -O 5,56 -E 4,1 -B 5
 --cs reference.fa contigs.fa | sort -k6,6 -k8,8n > alignments.paf
```

Variants were called on each haplotype using paftools (`https://github.com/lh3/minimap2/tree/master/misc`).

```
paftools.js call -L 50000 -f reference.fa alignments.paf > calls.vcf
```

We developed a pipeline for merging and filtering our variant calls, and to create a multisample VCF file representing a pangenome graph ("pangenome.vcf") based on our assembly-based callset ("variants.vcf"). Details can be found here: https://bitbucket.org/jana_ebler/genotyping-experiments/src/master/data/rules/assembly-vcfs.smk

7.2 Re-genotyping

Depending on which genotyping tool was run, we either directly genotyped the callset variants ("variants.vcf") or we used their pangenome graph representation ("pangenome.vcf"). We used the corresponding VCFs as input variants for the genotyping tools and genotyped them based on short Illumina reads as described in Section *Comparison to existing genotyping methods* of the main paper.

We ran BayesTyper (version v1.5) and PanGenie with default parameters using the raw, unaligned Illumina reads (FASTQ format) as input. For BayesTyper, we used the Snakemake pipeline provided in their repository (https://github.com/bioinformatics-centre/BayesTyper). PanGenie (https://github.com/eblerjana/pangenie, commit: `1f3d2d2`, using `jellyfish 2.2.10`) was run based on the command shown below,

```
PanGenie -i reads.fq -v pangenome.vcf -r reference.fa -o pangenie -j 24 -t 24 -g
```

The remaining tools were provided with the aligned reads in BAM format, produced by mapping them to the reference genome using bwa. Platypus (version 0.8.1) was run in re-typing mode with additional options `--source=variants.vcf`, `--minPosterior=0` and `--getVariantsFromBAMs=0` based on all callset variants ("variants.vcf").

In order to run GATK (version 4.1.3.0), we first marked duplicates in our BAMs and then used HaplotypeCaller in re-typing mode in order to compute genotypes for the input variants using the command below. Note that we did not genotype large variants with GATK, therefore we removed them from the input VCF file prior to genotyping.

```
GATK HaplotypeCaller
--reference reference.fa
---input reads.bam
---output gatk.vcf
---minimum-mapping-quality 20
---genotyping-mode GENOTYPE_GIVEN_ALLELES
---alleles variants_no_large.vcf
```

In order to run Paragraph (version v2a), we first computed the depth of the input BAM file using the command

```
/bin/idxdepth -b reads.bam -r reference.fasta -o depth.json
```

and prepared the Manifest file required for genotyping. In the next step, we used the command `bin/multigrmpy.py` with default parameters in order to genotype the input variants (pangenome graph representation). Note that we removed all variants shorter than 20 bp from the input VCF before running Paragraph in order to only type midsize and large variants.

GraphTyper (version 2.7.1) was run on all large callset variants ($\geq$ 50bp) using the following command:

```
graphtyper genotype_sv reference.fa variants_large.vcf --sam=reads.bam
--output=graphtyper
```

We ran the Giraffe genotyping pipeline using the Snakmake workflow provided here: https://github.com/vgteam/vg_snakemake (commit e2a60b, VG v1.30.0) using all large variants contained in our callset VCF ("variants.vcf").

7.3 Variant detection based on short reads

Besides re-genotyping our assembly-based variant calls using GATK and Platypus, we also ran both tools in discovery mode. For Platypus this was done based on the command:

```
Platypus callVariants --bamFiles=reads.bam --refFile=reference.fa
--output=platypus-calling.vcf
```

For GATK, we marked duplicates in our BAMs (as before) and called variants as:

```
GATK HaplotypeCaller
--reference reference.fa
--input reads.bam
--output gatk-calling.vcf
--minimum-mapping-quality 20
--genotyping-mode DISCOVERY
```

The complete pipeline used to run the evaluation including the commands used to run all tools can be found here:
https://bitbucket.org/jana_ebler/genotyping-experiments/src/master/genotyping/

References

1. Cleary, J. G. *et al.* Comparing variant call files for performance benchmarking of next-generation sequencing variant calling pipelines. *BioRxiv* 023754 (2015).
2. Pritt, J., Chen, N.-C. & Langmead, B. Forge: prioritizing variants for graph genomes. *Genome biology* **19**, 1–16 (2018).
3. Zook, J. M. *et al.* An open resource for accurately benchmarking small variant and reference calls. *Nat. biotechnology* **37**, 561 (2019).
4. Li, H. *et al.* A synthetic-diploid benchmark for accurate variant-calling evaluation. *Nat. methods* **15**, 595–597 (2018).
5. Dilthey, A. T. *et al.* HLA*LA-HLA typing from linearly projected graph alignments. *Bioinformatics* **35**, 4394–4396 (2019).
6. Robinson, J., Mistry, K., McWilliam, H., Lopez, R. & Marsh, S. G. E. IPD–the immuno polymorphism database. *Nucleic Acids Res.* **38**, D863–9 (2010).
7. Li, H. Minimap2: pairwise alignment for nucleotide sequences. *Bioinformatics* **34**, 3094–3100 (2018).
8. Abi-Rached, L. *et al.* Immune diversity sheds light on missing variation in worldwide genetic diversity panels. *PLoS One* **13**, e0206512 (2018).
9. Gourraud, P.-A. *et al.* HLA diversity in the 1000 genomes dataset. *PLoS One* **9**, e97282 (2014).
10. Dilthey, A. T. *et al.* High-Accuracy HLA type inference from Whole-Genome sequencing data using population reference graphs. *PLoS Comput. Biol.* **12**, e1005151 (2016).
11. Danecek, P. *et al.* The variant call format and vcftools. *Bioinformatics* **27**, 2156–2158 (2011).
12. Benjamini, Y. & Hochberg, Y. Controlling the false discovery rate: a practical and powerful approach to multiple testing. *J. Royal statistical society: series B (Methodological)* **57**, 289–300 (1995).
13. Wagner, J. *et al.* Towards a comprehensive variation benchmark for challenging medically-relevant autosomal genes. *bioRxiv* (2021).
14. Collins, R. L. *et al.* A structural variation reference for medical and population genetics. *Nature* **581**, 444–451 (2020).
15. DePristo, M. A. *et al.* A framework for variation discovery and genotyping using next-generation dna sequencing data. *Nat. genetics* **43**, 491 (2011).
16. Sherry, S. T. *et al.* dbsnp: the ncbi database of genetic variation. *Nucleic acids research* **29**, 308–311 (2001).
17. Buniello, A. *et al.* The nhgri-ebi gwas catalog of published genome-wide association studies, targeted arrays and summary statistics 2019. *Nucleic acids research* **47**, D1005–D1012 (2019).
18. Purcell, S. *et al.* Plink: a tool set for whole-genome association and population-based linkage analyses. *The Am. journal human genetics* **81**, 559–575 (2007).
19. ENCODE Project Consortium *et al.* The encode (encyclopedia of dna elements) project. *Science* **306**, 636–640 (2004).

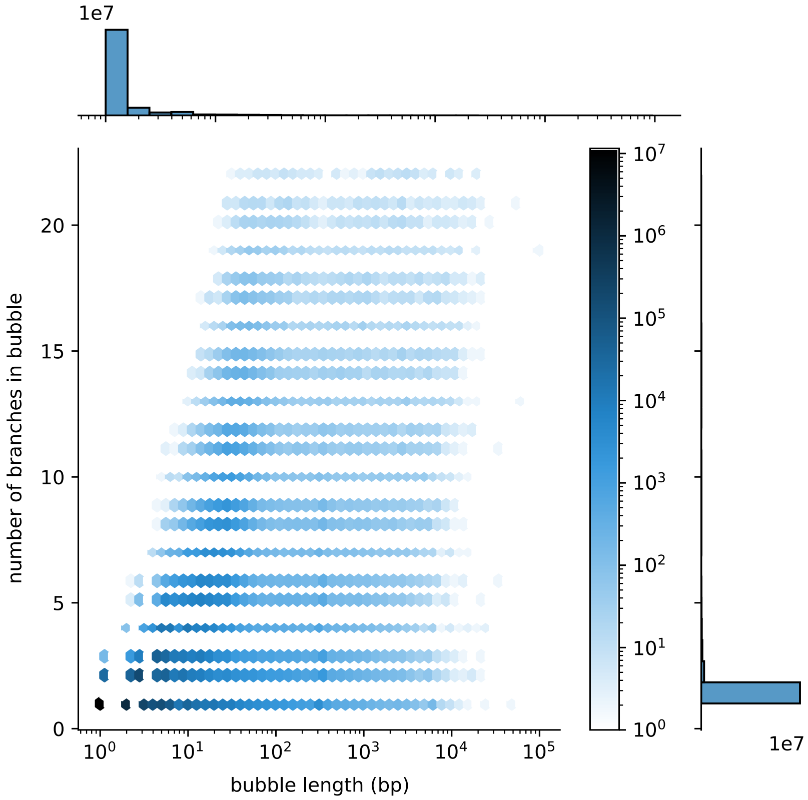

Supplementary Figure 1. Variant calling and graph construction. For our pangenome graph constructed from eleven samples, we show the number of branches in a bubble as a function of its length which we define by the length of the longest path through the bubble (in bp).

genotype concordance

	callset				
truth		0/0	0/1	1/1	./.
	0/0	T_0/0	F_0/0	F_0/0	
	0/1	T_0/1	T_0/1	F_0/1	
	1/1	F_1/1	F_1/1	T_1/1	
	./.				

precision

	callset				
truth		0/0	0/1	1/1	./.
	0/0		FP	FP	
	0/1		TP	FP	
	1/1		FP	TP	
	./.		FP	FP	

recall

	callset				
truth		0/0	0/1	1/1	./.
	0/0				
	0/1	FN	TP	FN	FN
	1/1	FN	FN	TP	FN
	./.				

Supplementary Figure 2. Metrics used to evaluate genotyping results and how they define errors.

a

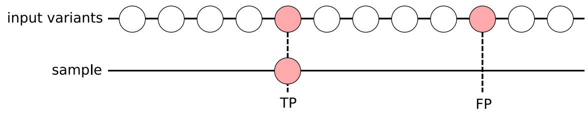

$$\text{precision} = \frac{1}{1+1} = 0.5$$

b

	**callset**				
**truth**		**0/0**	**0/1**	**1/1**	**./.**
	**0/0**	9298	374	41	14
	**0/1**	125	564	35	0
	**1/1**	8	49	247	2
	**./.**	0	0	0	0

$$\text{precision} = \frac{564+247}{564+247+374+41+35+49} = 0.62$$

$$\text{recall} = \frac{564+247}{564+247+125+8+35+2+49} = 0.79$$

$$\text{genotype concordance} = \frac{9298}{9298+374+41} + \frac{564}{564+125+35} + \frac{247}{247+49+8} = 0.85$$

Supplementary Figure 3. Evaluation example. a) Shown is a set of variants (represented by circles) that are used as input for genotyping and a sample carrying only one of these variants (i.e. the true genotype for all others is 0/0). Both variants colored in red were genotyped as non-0/0, resulting in a genotyping error for the second one which will be counted as a false positive call. This example illustrates why precision/recall measures are not ideal for a re-genotyping scenario: when variants are called across a large set of samples and then genotyped in another, new sample, there will be a large fraction of variants absent in the new sample. Thus, even if the false positive rate is low, the number of false positives will increase as the number of input variants increases, while the number of true positives is limited. In this example, only a single genotyping error will decrease the precision to 0.5. **b)** Confusion matrix for PanGenie and the resulting values for precision, recall and weighted genotype concordance (matrix for large insertions in repetitive regions). While the false positive rate is low (only 415 of 9713 variants absent from the truth set are genotyped as non-0/0), it is relatively high compared to the number of true positives which explains why the precision is so low compared to the weighted genotype concordance. The same effect applies to all other re-genotyping methods.

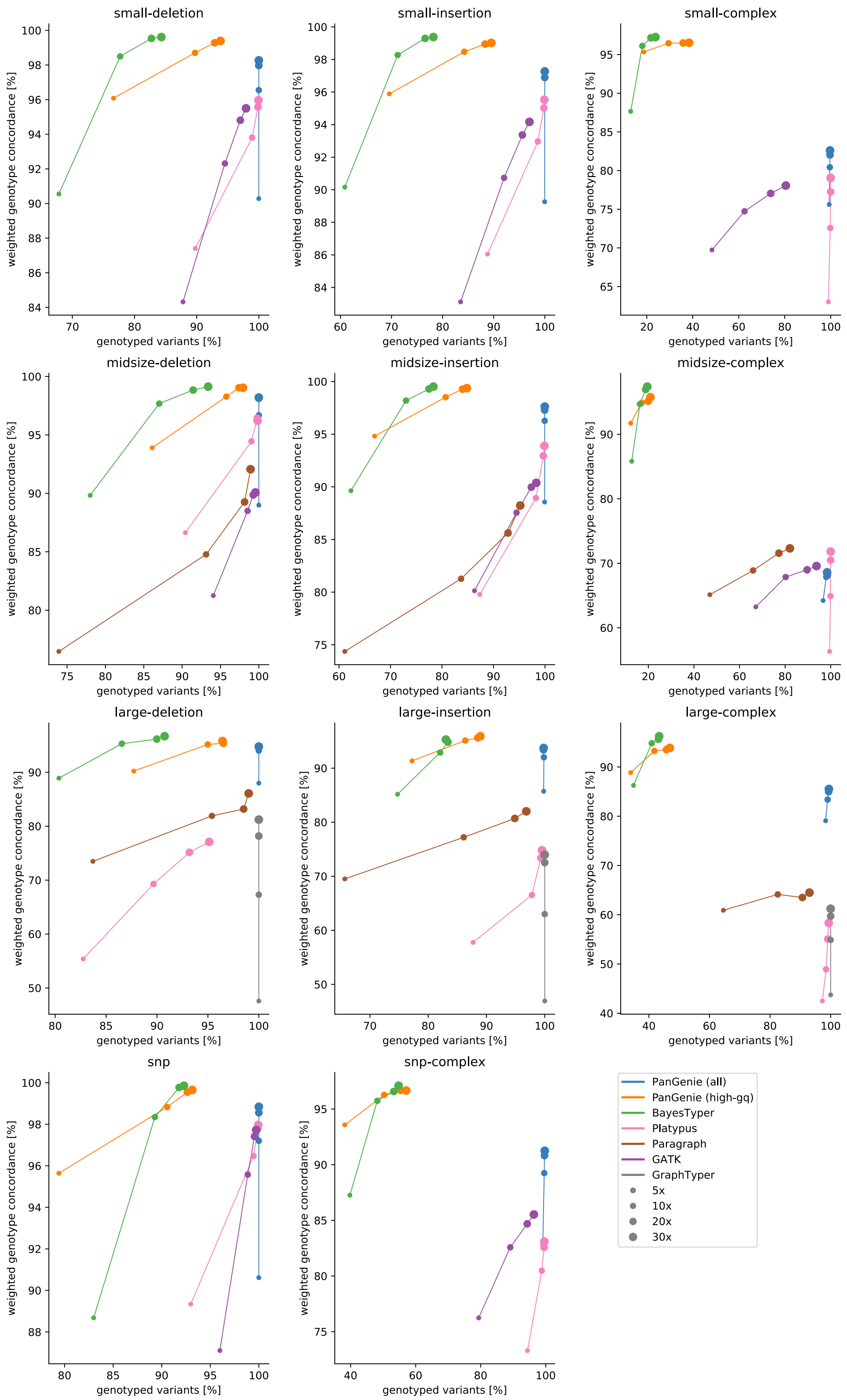

Supplementary Figure 4. weighted genotype concordance for NA24385 (non-repetitive regions). Weighted genotype concordance at different coverages for sample NA24385. We ran PanGenie, BayesTyper, Paragraph, Platypus, GATK, GraphTyper and Giraffe in order to re-genotype all callset variants. Besides not applying any filter on the reported genotype qualities (“all”), we additionally report genotyping statistics for PanGenie when using “high-gq” filtering (genotype quality $>= 200$). SNPs, insertions and deletions include all respective variants in biallelic regions of the genome, while *complex* contains all variant alleles falling into regions with complex bubbles in the pangenome graph representation.

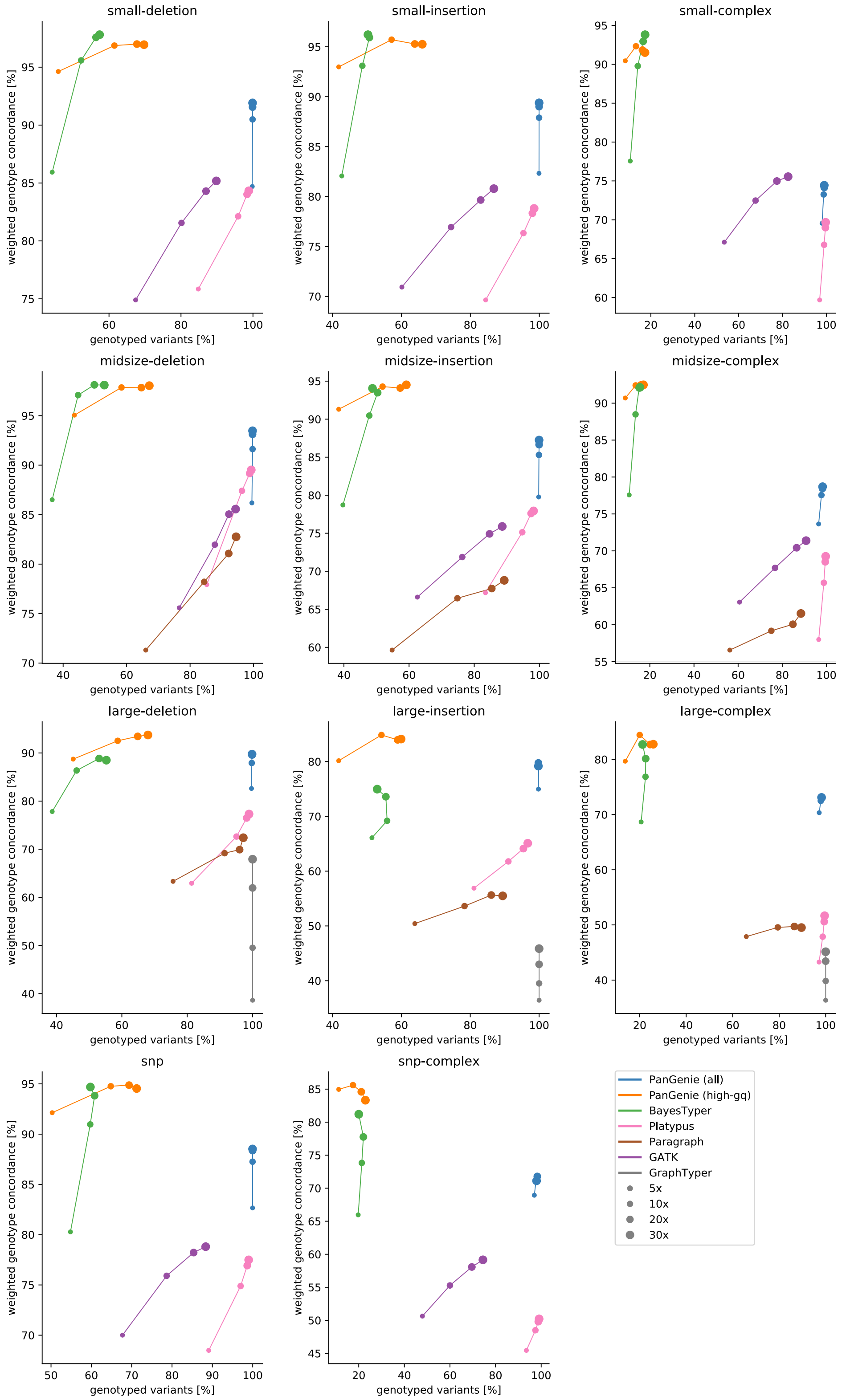

Supplementary Figure 5. weighted genotype concordance for NA24385 (STR/VNTR regions). Weighted genotype concordance at different coverages for sample NA24385. We ran PanGenie, BayesTyper, Paragraph, Platypus, GATK, GraphTyper and Giraffe in order to re-genotype all callset variants. Besides not applying any filter on the reported genotype qualities (“all”), we additionally report genotyping statistics for PanGenie when using “high-gq” filtering (genotype quality >= 200). SNPs, insertions and deletions include all respective variants in biallelic regions of the genome, while *complex* contains all variant alleles falling into regions with complex bubbles in the pangenome graph representation.

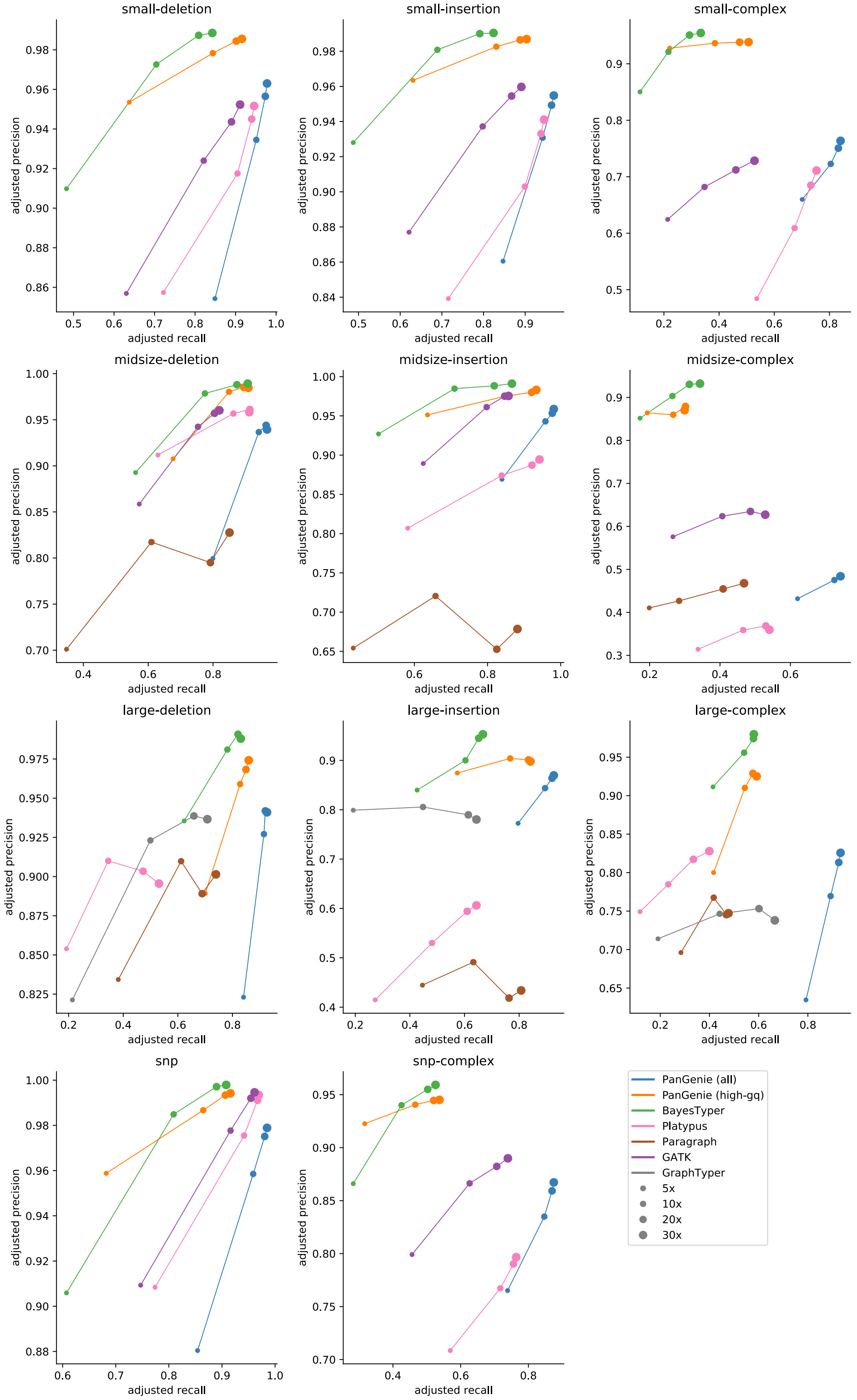

Supplementary Figure 6. adjusted precision/recall for NA24385 (non-repetitive regions). Adjusted precision/recall at different coverages for sample NA24385. We ran PanGenie, BayesTyper, Paragraph, Platypus, GATK, GraphTyper and Giraffe in order to re-genotype all callset variants. Besides not applying any filter on the reported genotype qualities ("all"), we additionally report genotyping statistics for PanGenie when using "high-gq" filtering (genotype quality >= 200). SNPs, insertions and deletions include all respective variants in biallelic regions of the genome, while *complex* contains all variant alleles falling into regions with complex bubbles in the pangenome graph representation.

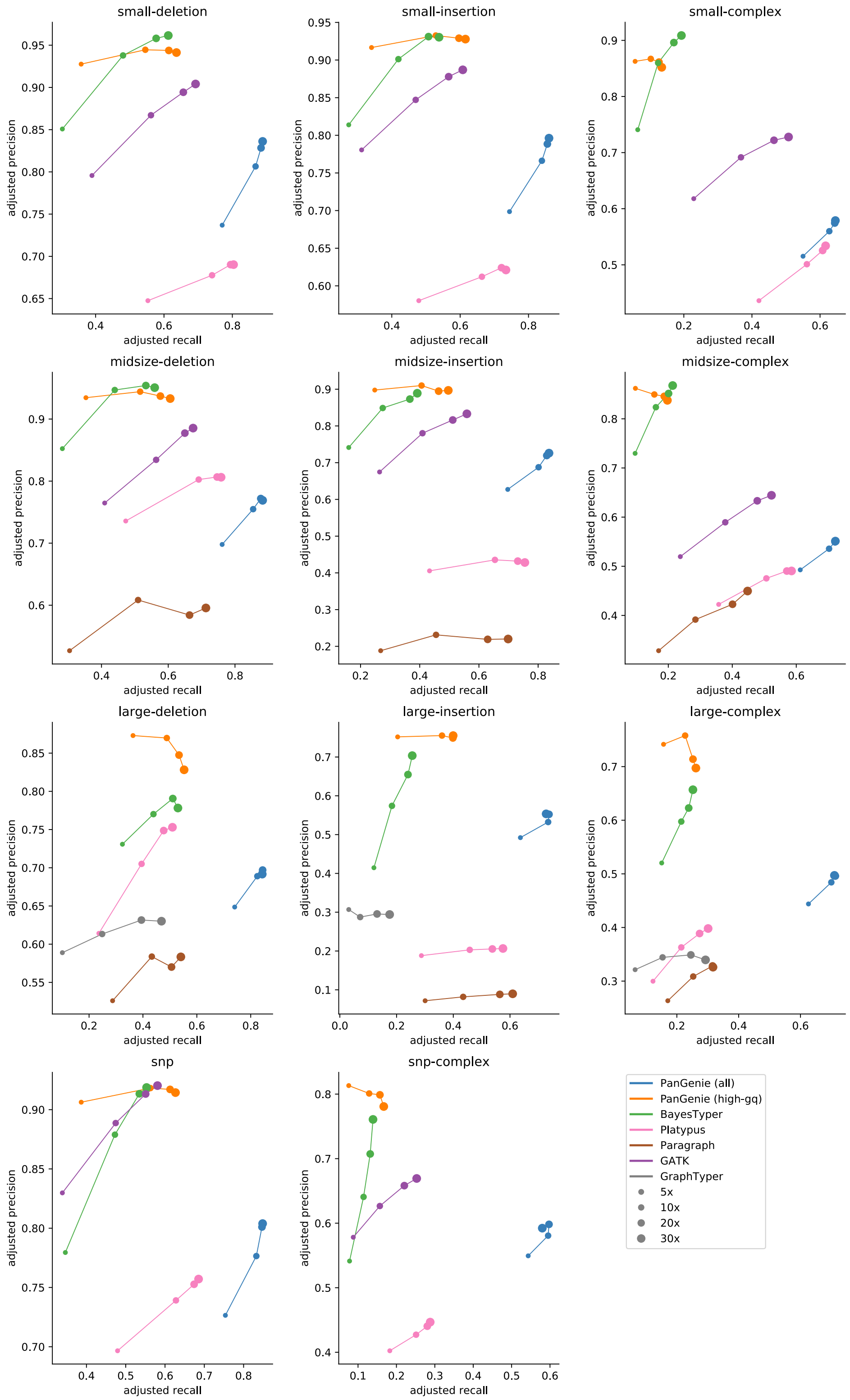

Supplementary Figure 7. adjusted precision/recall for NA24385 (STR/VNTR regions). Adjusted precision/recall at different coverages for sample NA24385. We ran PanGenie, BayesTyper, Paragraph, Platypus, GATK, GraphTyper and Giraffe in order to re-genotype all callset variants. Besides not applying any filter on the reported genotype qualities ("all"), we additionally report genotyping statistics for PanGenie when using "high-gq" filtering (genotype quality $>= 200$). SNPs, insertions and deletions include all respective variants in biallelic regions of the genome, while *complex* contains all variant alleles falling into regions with complex bubbles in the pangenome graph representation.

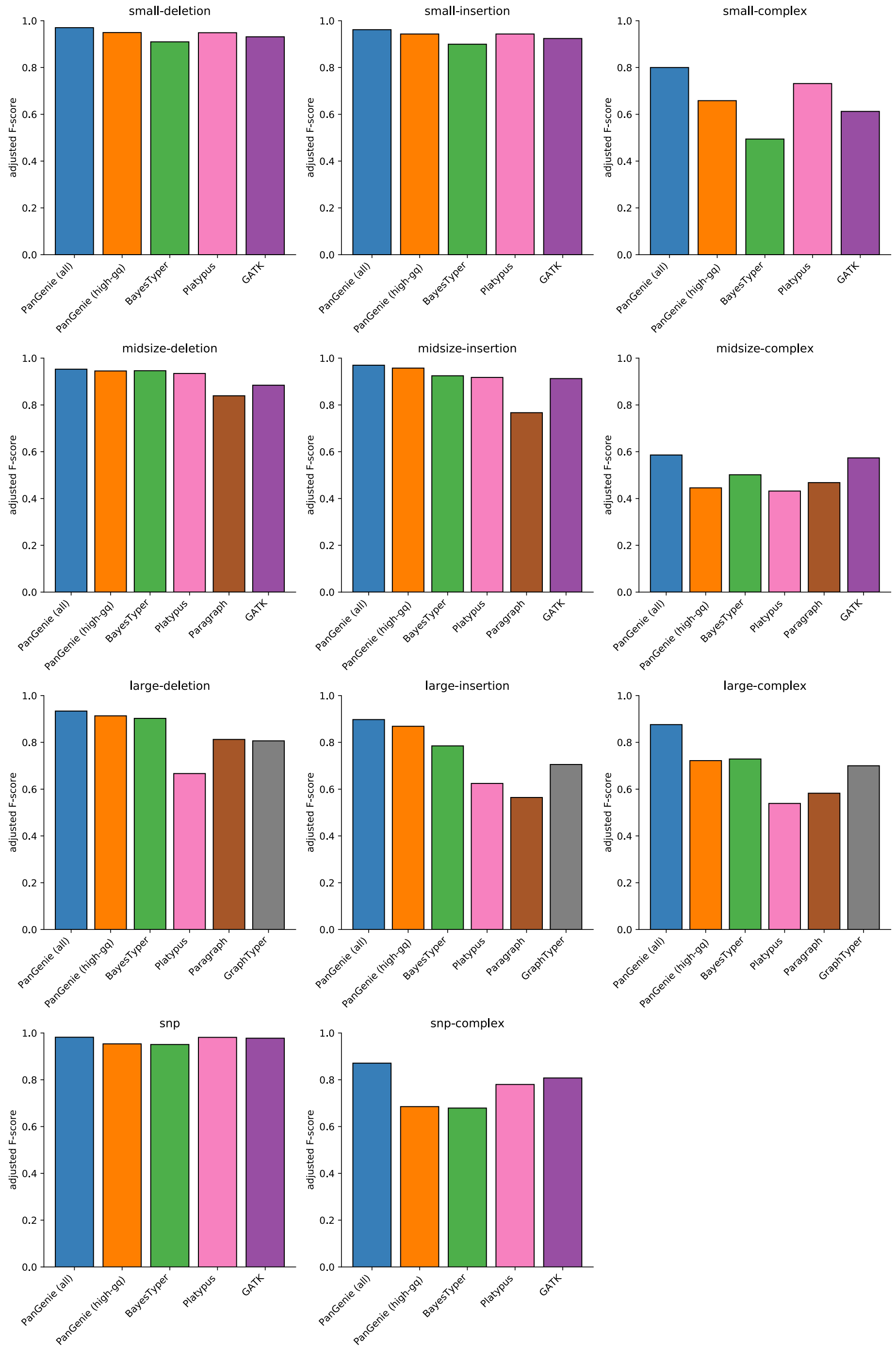

Supplementary Figure 8. adjusted F-score for NA24385 (non-repetitive regions). Adjusted F-score at coverage 30× for sample NA24385. We ran PanGenie, BayesTyper, Paragraph, Platypus, GATK, GraphTyper and Giraffe in order to re-genotype all callset variants. Besides not applying any filter on the reported genotype qualities ("all"), we additionally report genotyping statistics for PanGenie when using "high-gq" filtering (genotype quality $>= 200$). SNPs, insertions and deletions include all respective variants in biallelic regions of the genome, while *complex* contains all variant alleles falling into regions with complex bubbles in the pangenome graph representation.

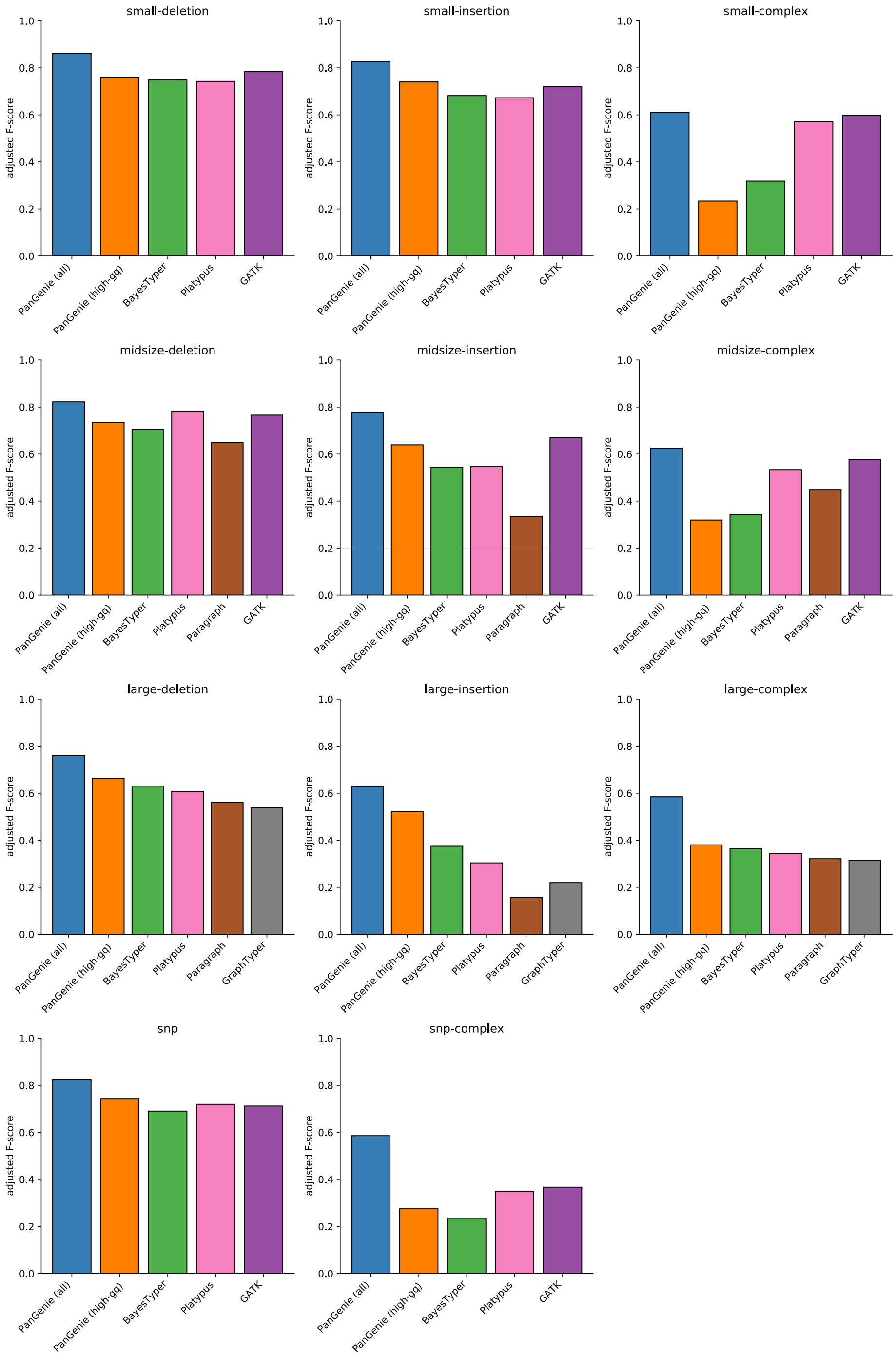

Supplementary Figure 9. adjusted F-score for NA24385 (STR/VNTR regions). Adjusted F-score at coverage 30× for sample NA24385. We ran PanGenie, BayesTyper, Paragraph, Platypus, GATK, GraphTyper and Giraffe in order to re-genotype all callset variants. Besides not applying any filter on the reported genotype qualities ("all"), we additionally report genotyping statistics for PanGenie when using "high-gq" filtering (genotype quality >= 200). SNPs, insertions and deletions include all respective variants in biallelic regions of the genome, while *complex* contains all variant alleles falling into regions with complex bubbles in the pangenome graph representation.

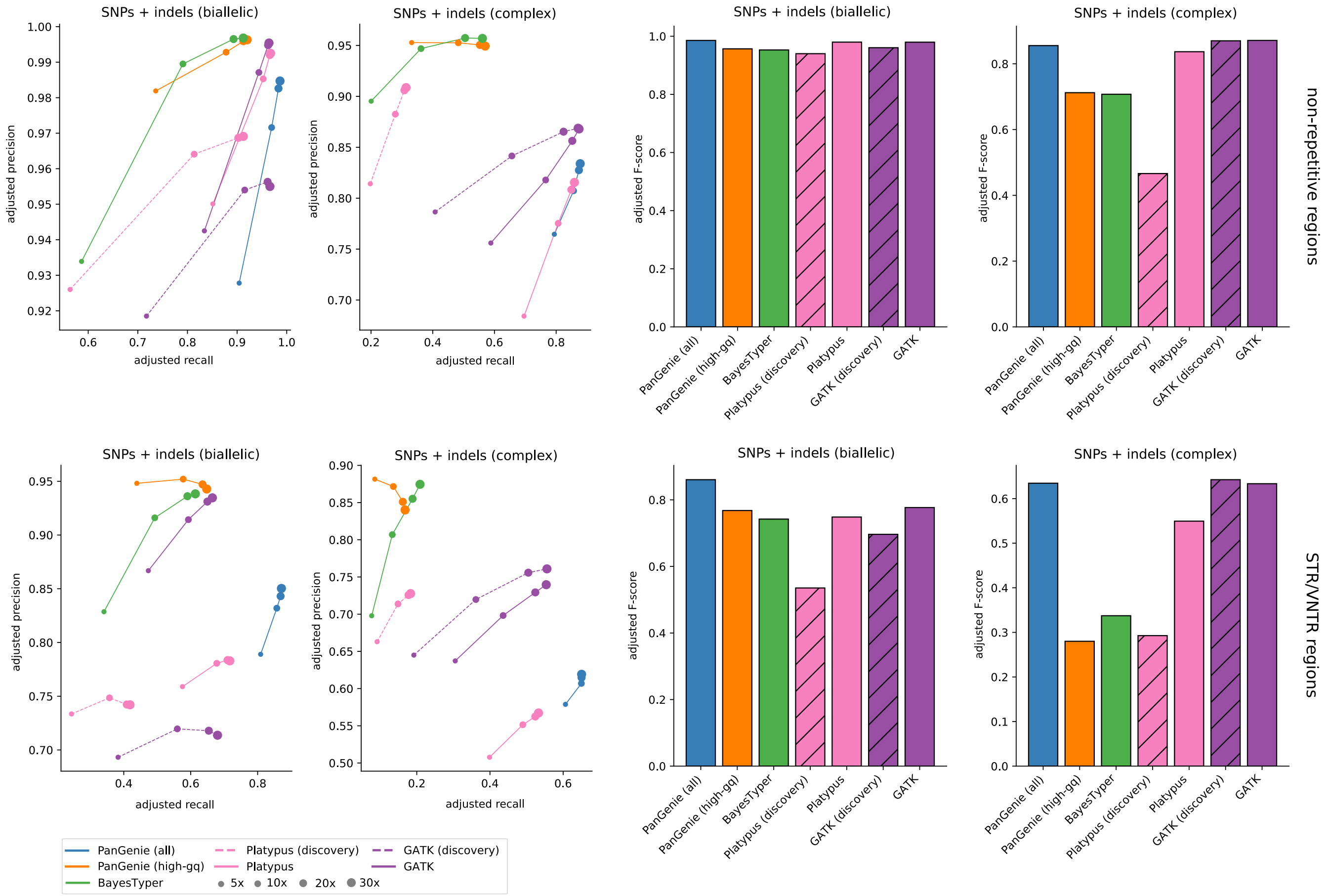

Supplementary Figure 10. **variant discovery vs. re-genotyping for NA12878.** In addition to re-genotyping given variants, GATK and Platypus were run in discovery mode to detect and genotype their own SNPs and indels (< 50bp). Results were evaluated inside of STR/VNTR regions and in non-repetitive regions. Adjusted F-scores were computed for coverage level 30×. We separately evaluate results for all variants falling into biallelic and complex regions of the genome as defined by the bubble structures in the pangenome graph.

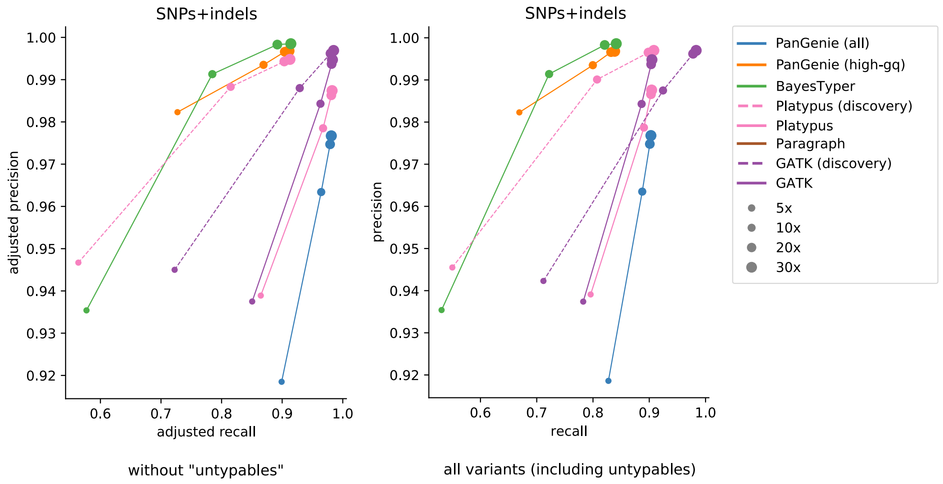

Supplementary Figure 11. **Comparison to GIAB small variants for NA12878.** The GIAB small variants benchmark set[3] was used as ground truth for evaluating the results of our "leave-one-out" experiment for SNPs and indels (< 50bp). We computed the adjusted precision and recall (left), as well as the un-adjusted versions (right) including variants unique to NA12878 and thus not genotypable by a re-genotyping approach. GATK and Platypus were additionally run in detection mode.

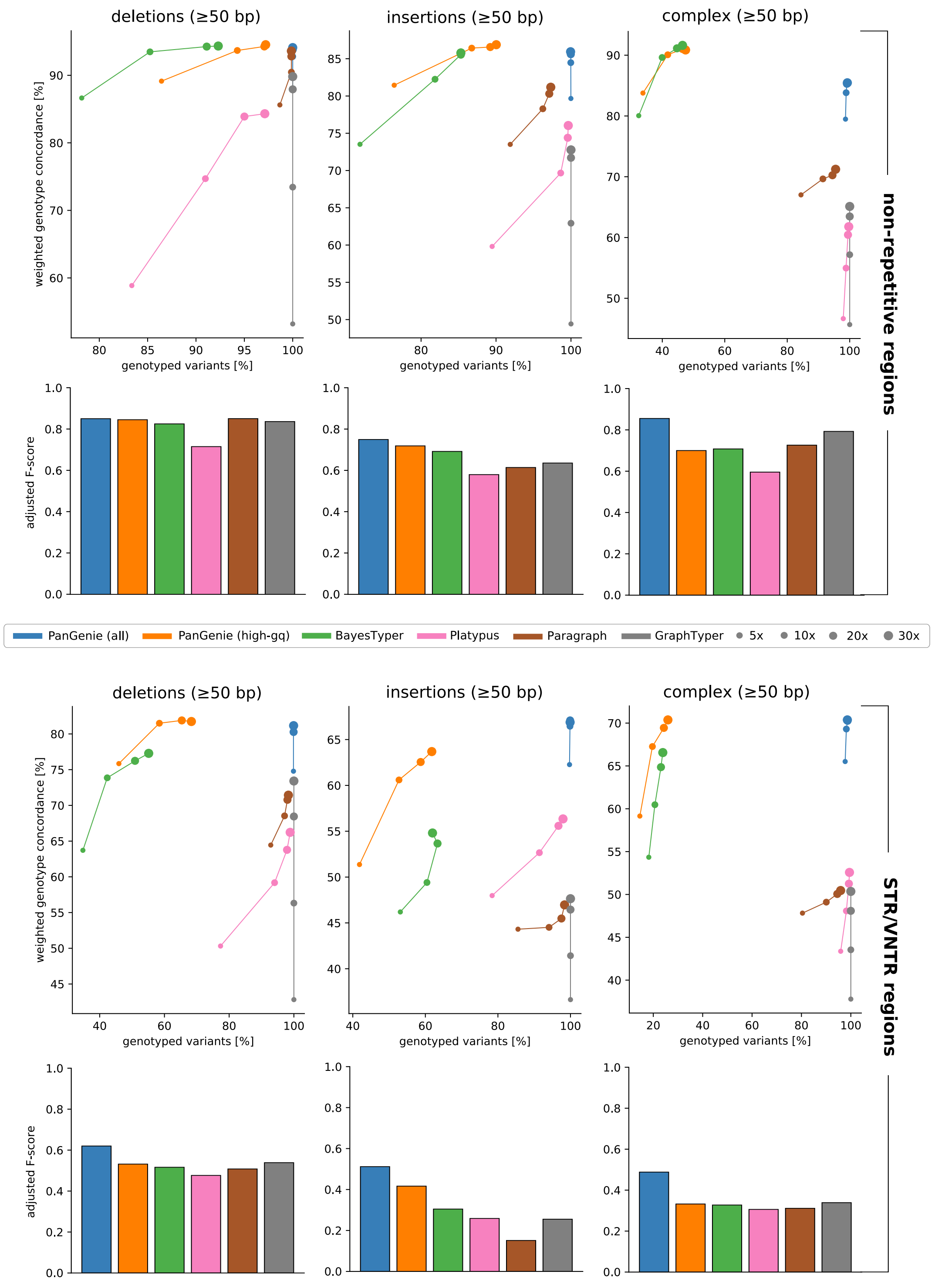

Supplementary Figure 12. Comparison to syndip benchmark SVs. SVs contained in the syndip benchmark set were used as ground truth for evaluation. We computed the weighted genotype concordance and the adjusted precision and recall metrics to evaluate our results.

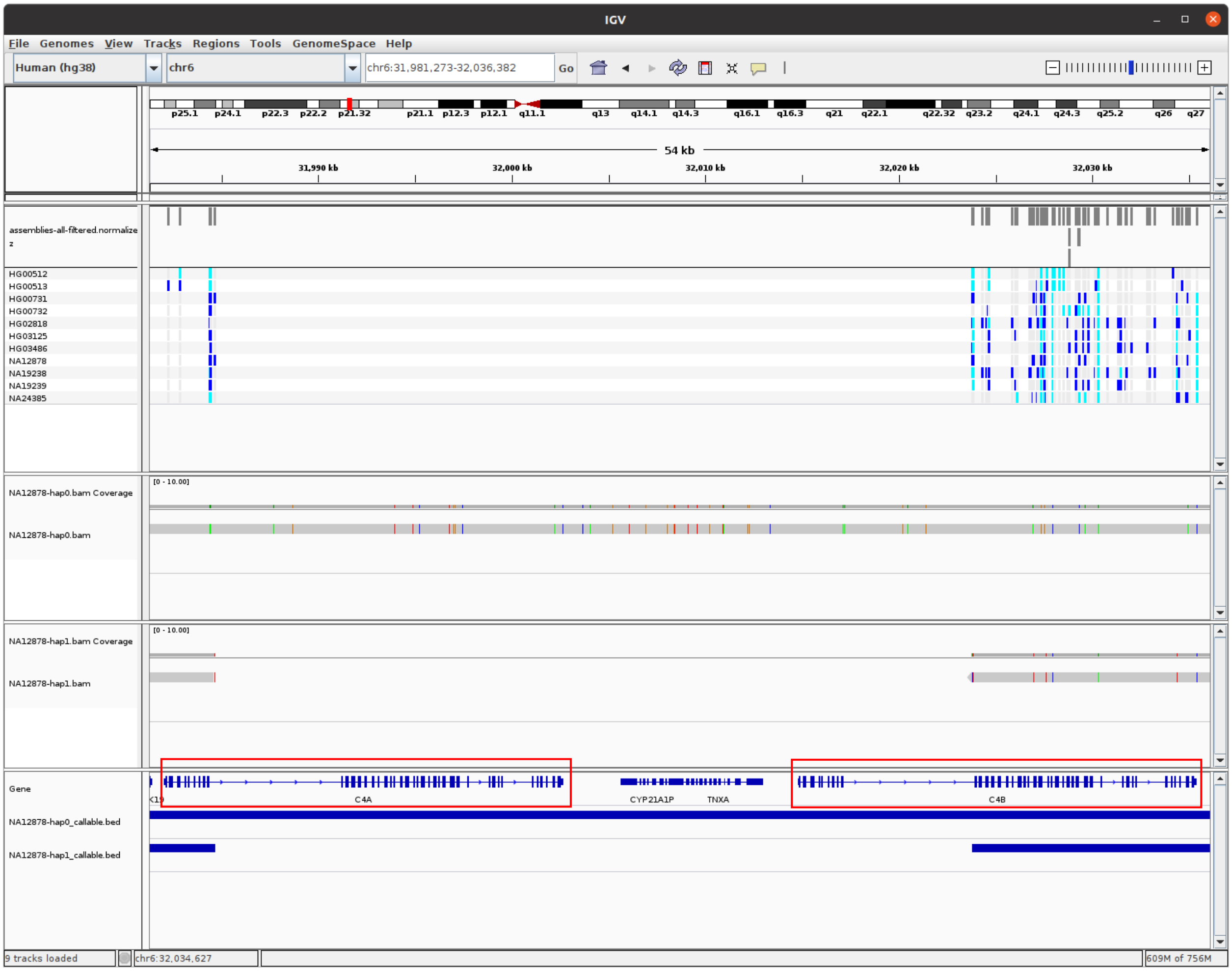

Supplementary Figure 13. **HLA genotyping**. While all other genes considered where fully covered by assembly alignment and therefore accessible for variant calling, the C4 genes where not since there was a large gap in the alignment of one haplotype of NA12878 (possibly caused by a large deletion) and the alignments of many other samples. Thus, the evaluation shown in a) corresponds only for the parts accessible by variant calling.

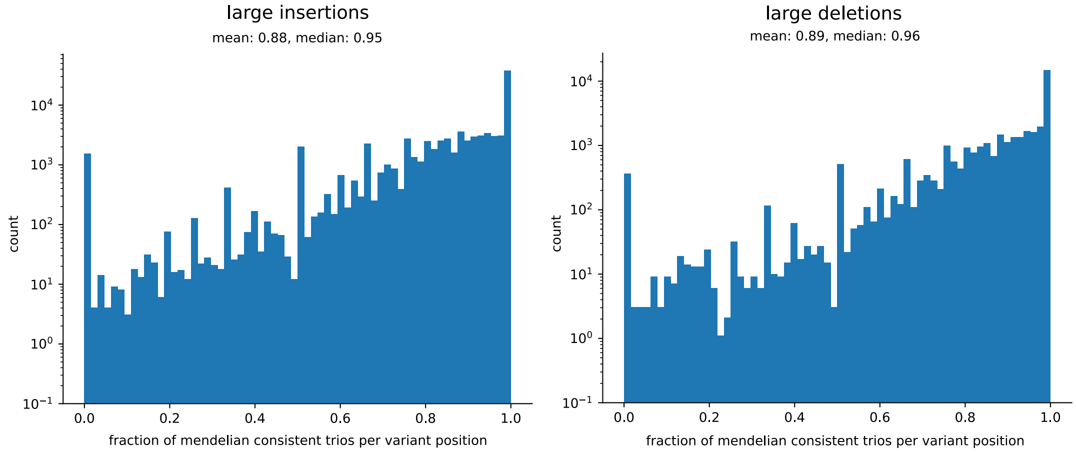

Supplementary Figure 14. **Mendelian Consistency.** Distribution of mendelian consistencies computed for each variant across all trios with at least two different genotypes. Our definition of mendelian consistency only takes trios with at least two different genotypes into consideration. That is, we exclude trios with all 0/0, 0/1 or 1/1 genotypes.

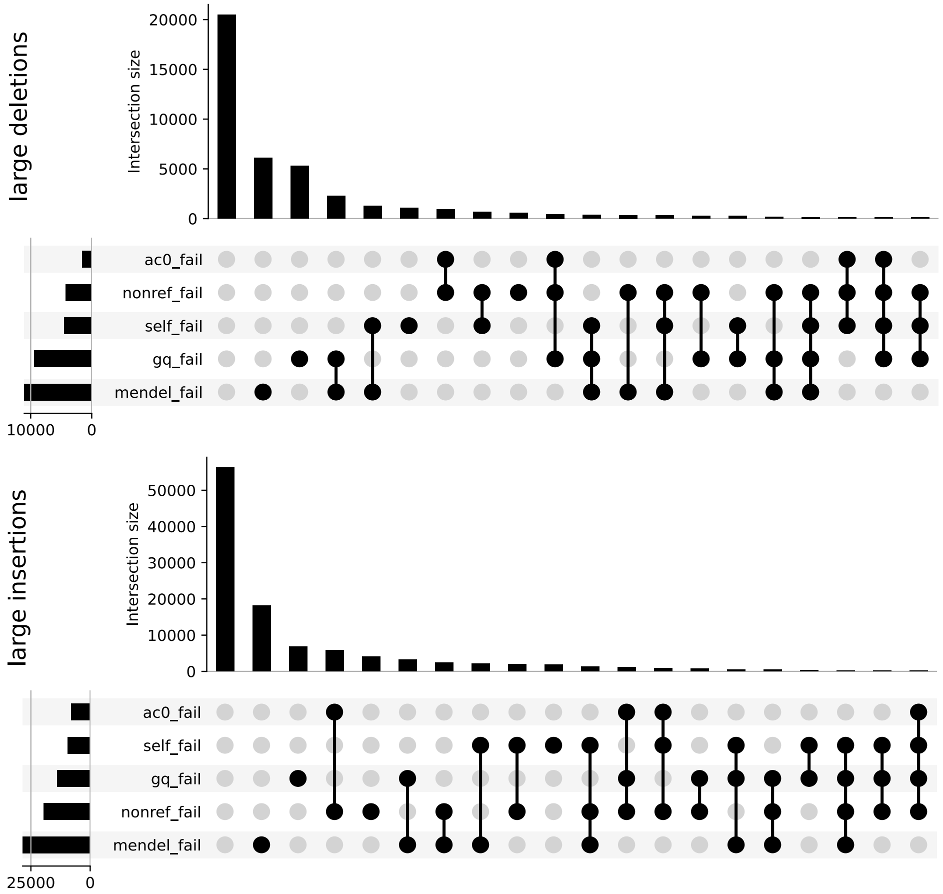

Supplementary Figure 15. Filters. We show all combinations of filters that we have applied to our genotyped variant callset and the respective number of variants in each subset. The black dots indicate that the respective filter failed.

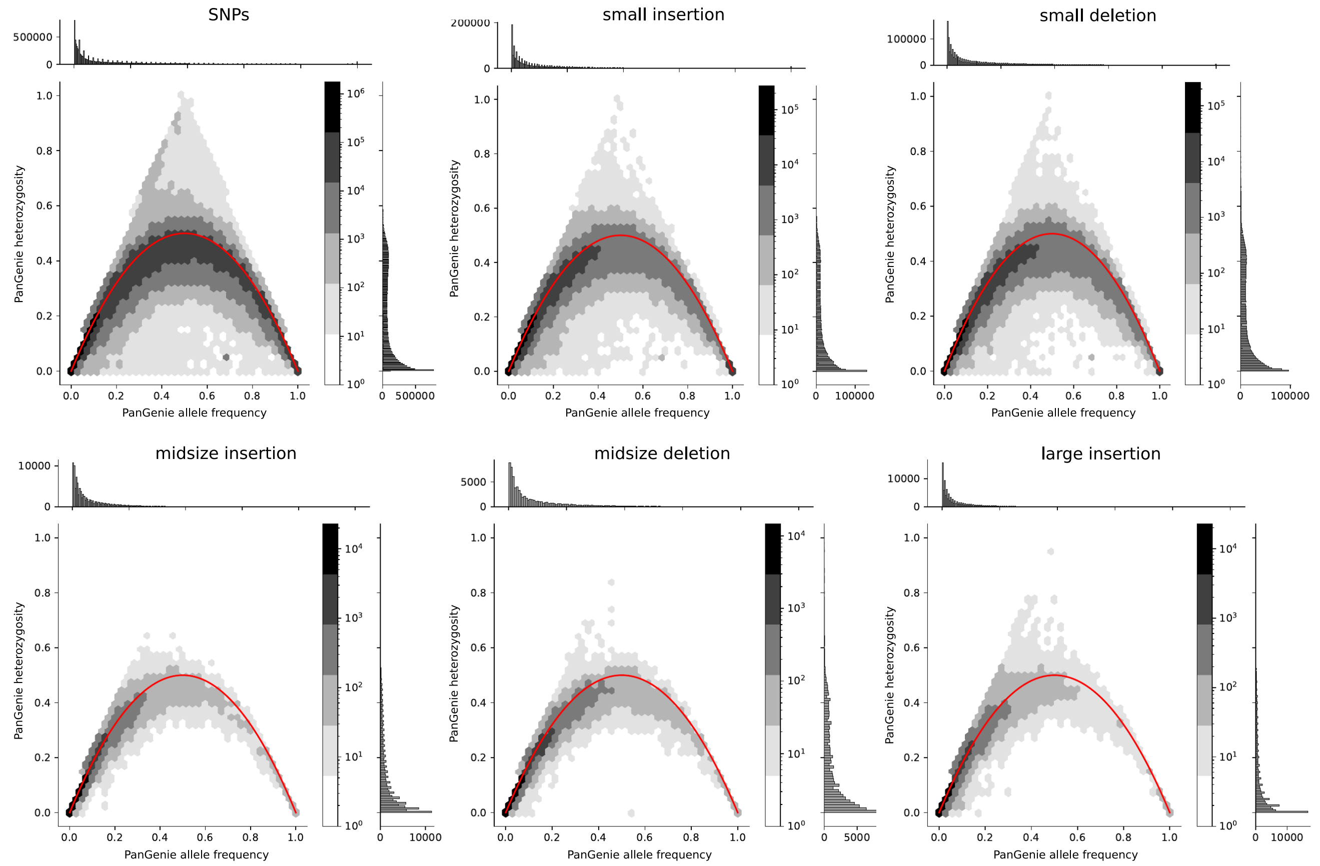

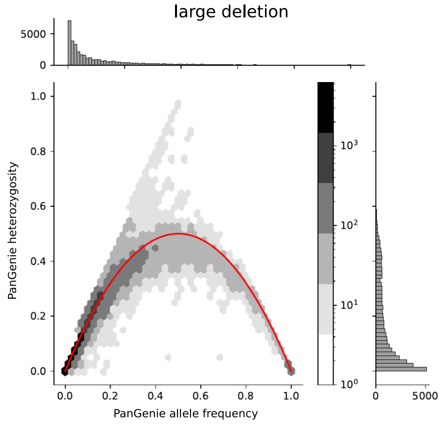

region	variant type	total	in HWE	
non-repetitive regions	SNPs	11,107,577	9,811,887	(88.3%)
	small deletions	1,009,997	882,871	(87.4%)
	small insertions	1,295,561	1,134,144	(87.5%)
	midsize deletions	6,567	5,856	(89.2%)
	midsize insertions	32,030	29,092	(90.8%)
	large deletions	5,272	4,624	(87.7%)
	large insertions	24,862	22,387	(90.0%)
repetitive regions	SNPs	762,222	636,904	(83.6%)
	small deletions	522,727	458,396	(87.7%)
	small insertions	288,992	251,756	(87.1%)
	midsize deletions	42,477	37,237	(87.7%)
	midsize insertions	37,601	33,699	(89.6%)
	large deletions	22,117	18,714	(84.6%)
	large insertions	50,876	45,093	(88.6%)

Supplementary Figure 16. Unfiltered set. Allele frequency vs. heterozygosity of the PanGenie genotypes across all 200 unrelated trio samples and all 11 panel samples for the unfiltered set of variants. The table shows the number of variants for which no significant deviation from Hardy-Weinberg equilibrium was observed.

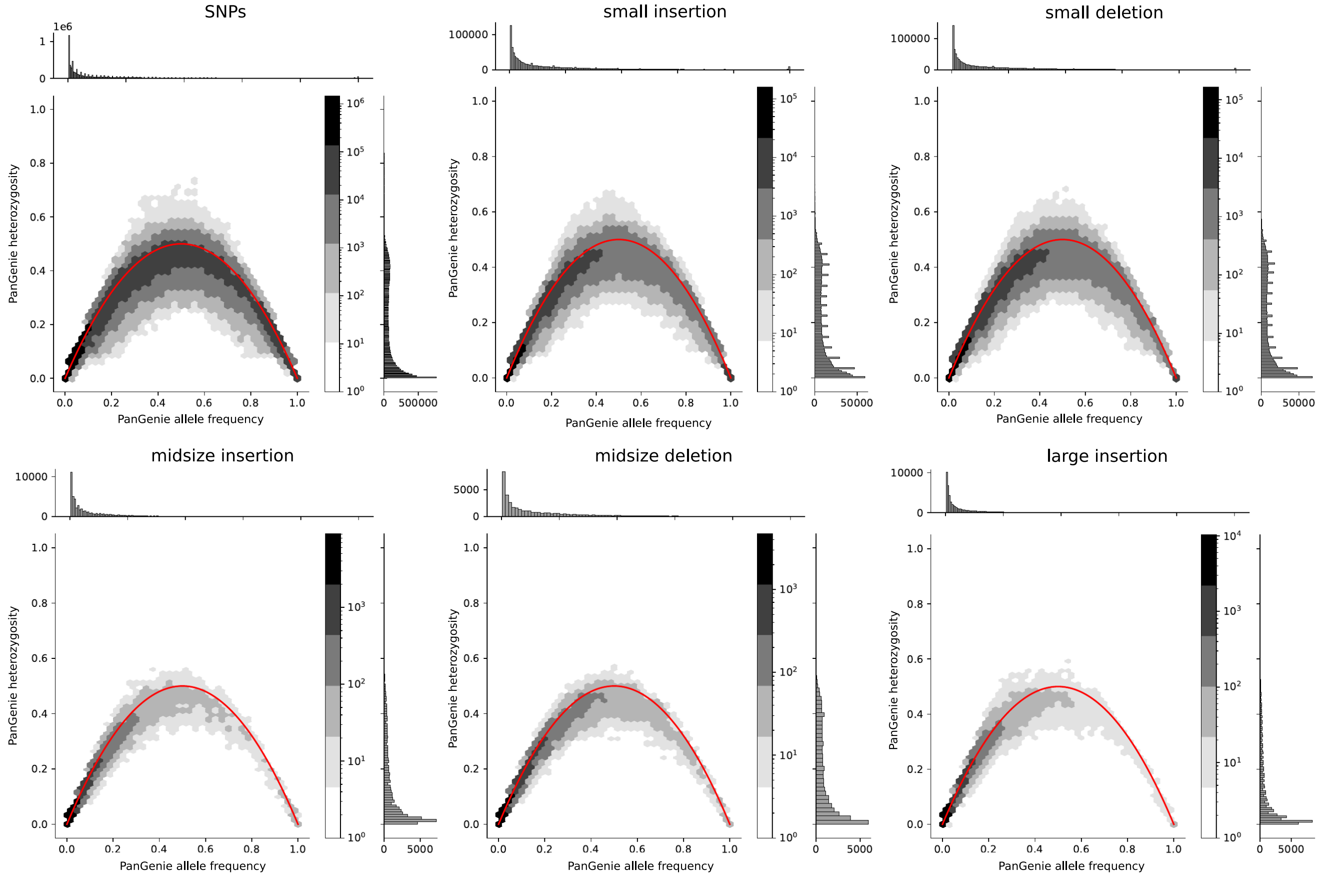

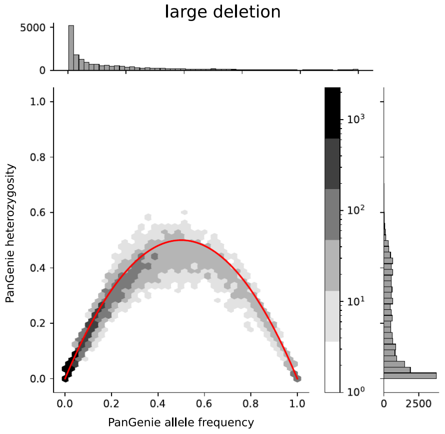

region	variant type	total	in HWE	
non-repetitive regions	SNPs	10,629,203	9,510,474	(89.5%)
	small deletions	863,268	768,534	(89.0%)
	small insertions	946,454	841,829	(89.0%)
	midsize deletions	6,259	5,676	(90.7%)
	midsize insertions	19,897	18,173	(91.3%)
	large deletions	4,425	3,913	(88.4%)
	large insertions	18,229	16,870	(92.5%)
repetitive regions	SNPs	542,171	483,116	(89.1%)
	small deletions	281,952	252,120	(89.4%)
	small insertions	182,362	163,843	(89.8%)
	midsize deletions	24,453	21,801	(89.1%)
	midsize insertions	22,823	20,897	(91.6%)
	large deletions	11,661	10,516	(90.2%)
	large insertions	27,351	25,481	(93.2%)

Supplementary Figure 17. Strict set. Allele frequency vs. heterozygosity of the PanGenie genotypes across all 200 unrelated trio samples and all 11 panel samples for the strictly filtered set of variants. The table shows the number of variants for which no significant deviation from Hardy-Weinberg equilibrium was observed.

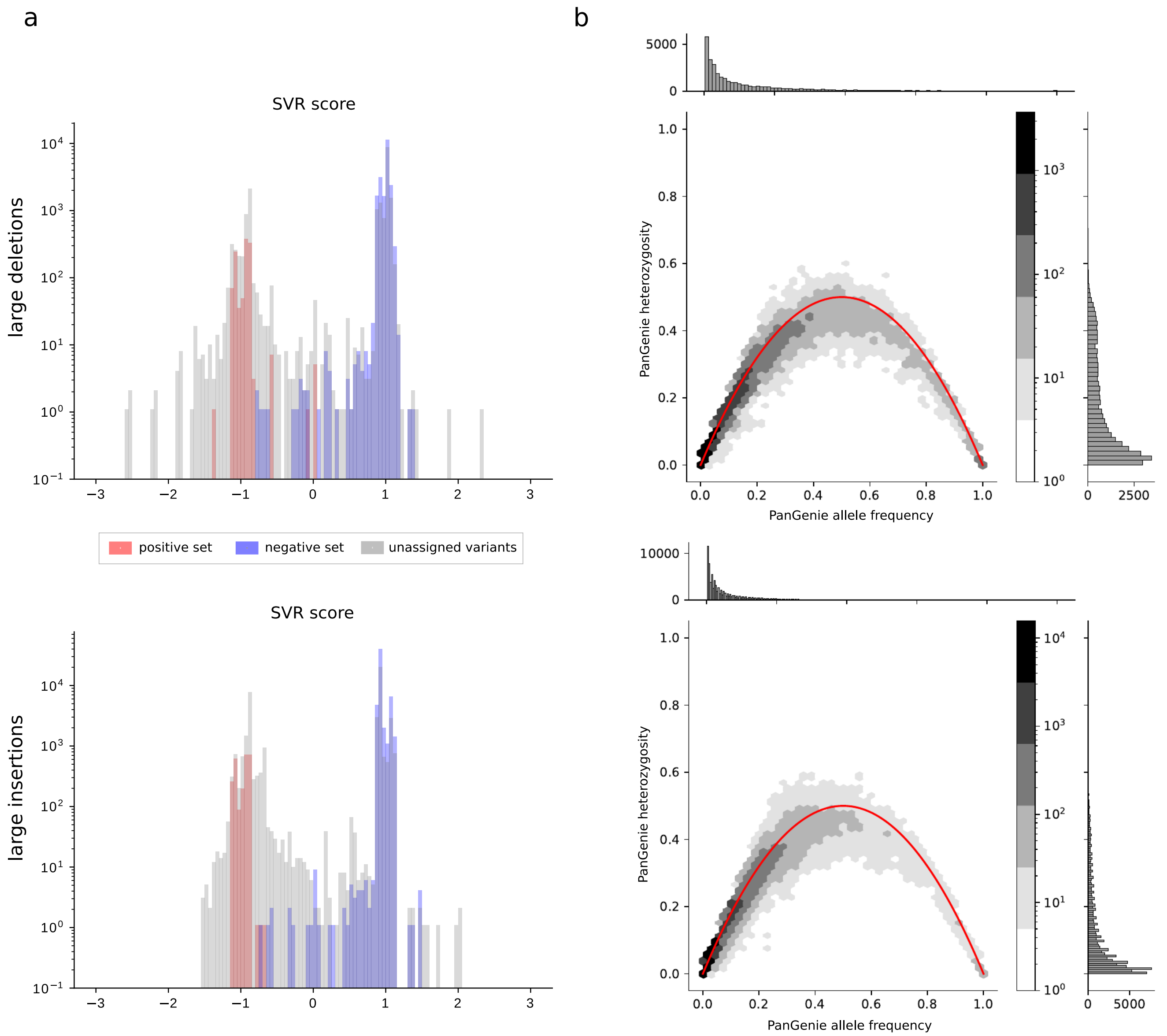

Supplementary Figure 18. **Lenient set. a)** Distributions of SVR scores predicted for the positive (blue), negative (red) and unassigned variants (grey). **b)** Allele frequency vs. heterozygosity of the PanGenie genotypes across all 200 unrelated trio samples and all 11 panel samples for the lenient set of variants. The lenient set contains all variants contained in the strict set (=positive set), as well as all variants for which the SVR score was ≥ -0.5.

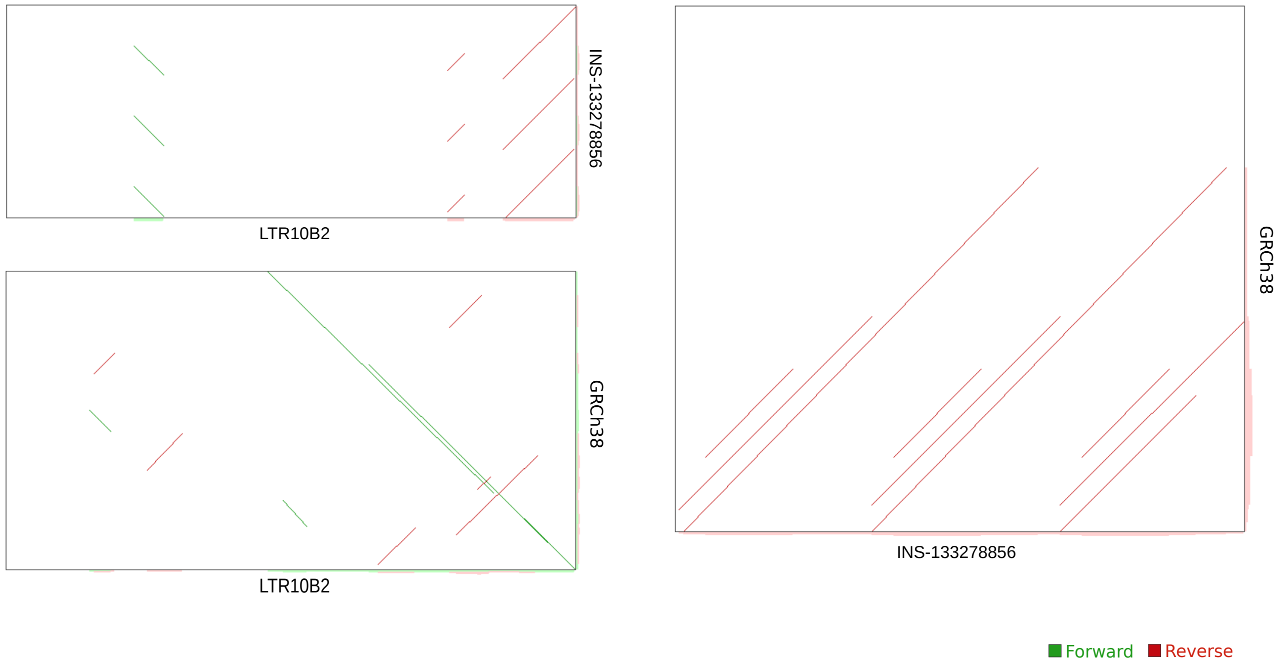

Supplementary Figure 19. LD analysis. Pairwise dot plots of the insertion sequence, LTR10B2 consensus sequence and the reference sequence of this region (GRCh38).

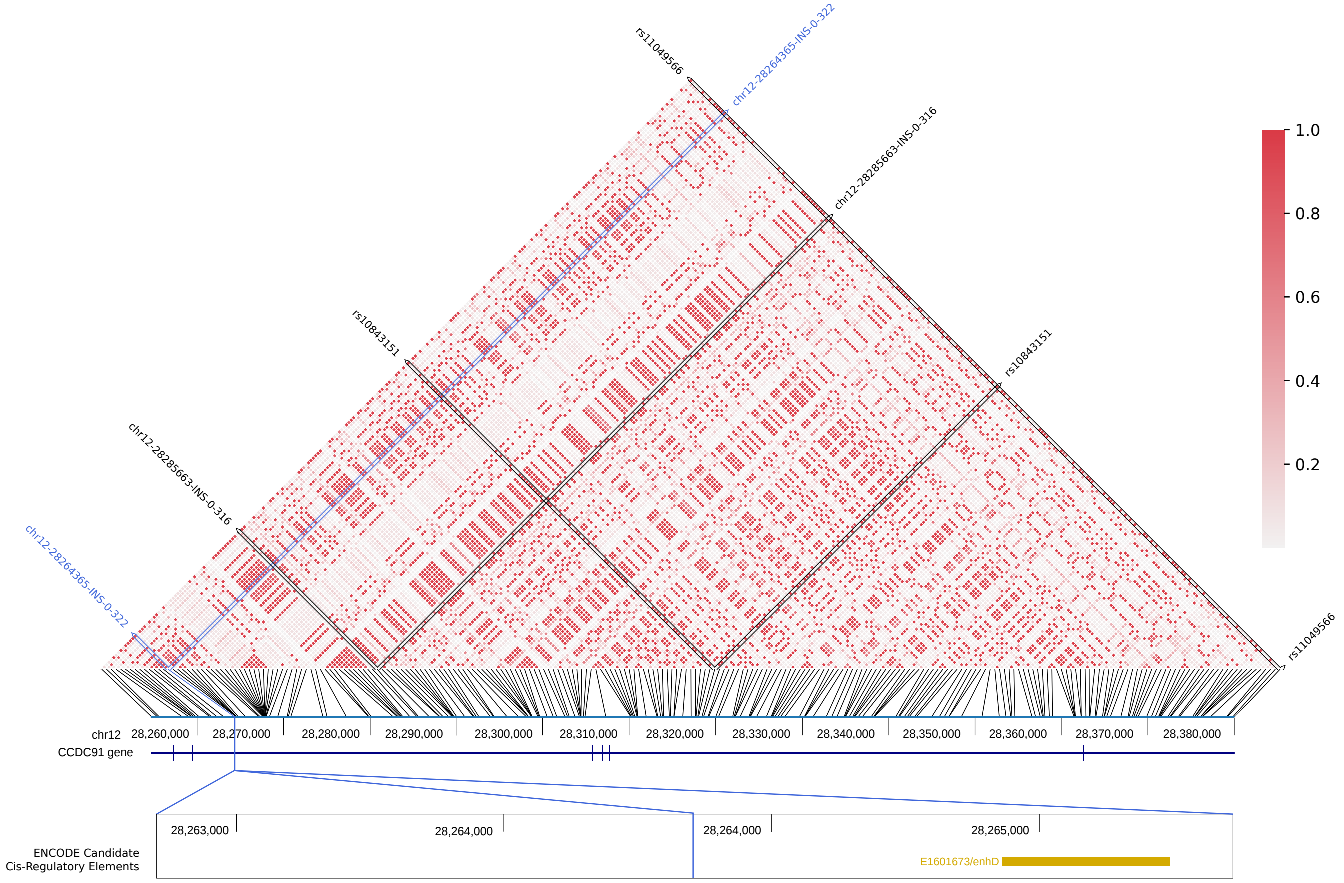

Supplementary Figure 20. LD analysis. We calculated LD for GWAS variants and SVs that were part of our assembly based callset. We detected an insertion (marked in blue) in CCDC91 gene which was in linkage disequilibrium with two GWAS SNPs (rs10843151 and rs11049566). The plots shows all callset variants with AF$\geq$ 0.05 in this region, GWAS variants are annotated with their name.

Sample	Haplotype	v13 / hifiasm			
		ctg. N50	# contigs	length (bp)	largest contig (bp)
HG00512	h1	34,301,582	1,777	3,188,143,530	101,334,844
HG00512	h2	34,874,650	1,449	3,163,234,783	112,440,880
HG00513	h1	45,364,295	1,500	3,136,967,952	133,503,586
HG00513	h2	45,319,314	1,227	3,113,449,797	139,638,253
HG00514	h1	17,395,387	1,983	3,141,391,447	94,121,208
HG00514	h2	18,722,440	1,650	3,121,917,894	72,949,789
HG00731	h1	35,342,509	2,218	3,179,873,135	130,295,053
HG00731	h2	31,517,176	1,791	3,145,395,507	131,214,380
HG00732	h1	22,794,071	1,084	3,161,371,445	70,183,886
HG00732	h2	18,785,010	849	3,128,313,934	85,401,373
HG00733	h1	32,098,695	1,711	3,141,624,750	81,981,905
HG00733	h2	39,889,742	1,327	3,127,483,091	110,942,518
HG02818	h1	14,735,342	1,645	3,148,193,201	62,389,557
HG02818	h2	13,891,585	1,346	3,131,545,528	53,330,289
HG03125	h1	19,740,003	1,453	3,144,381,367	78,085,250
HG03125	h2	16,030,141	1,233	3,121,673,094	69,854,835
HG03486	h1	13,742,469	1,400	3,172,408,948	63,278,388
HG03486	h2	15,627,668	1,269	3,152,826,116	58,336,852
NA12878	h1	33,400,276	3,631	3,129,308,283	104,882,848
NA12878	h2	27,880,200	3,014	3,108,352,699	110,737,365
NA19238	h1	15,612,125	2,954	3,126,238,496	71,400,915
NA19238	h2	15,239,724	2,418	3,107,339,654	72,441,052
NA19239	h1	19,056,746	2,217	3,198,825,166	84,398,966
NA19239	h2	16,698,371	1,806	3,171,600,679	95,184,935
NA19240	h1	29,153,232	1,978	3,153,890,228	104,206,385
NA19240	h2	32,117,261	1,588	3,136,752,685	95,070,688
NA24385	h1	23,950,673	1,649	3,173,344,587	98,025,482
NA24385	h2	28,576,363	1,306	3,156,300,763	111,314,854
mean		25,423,466	1,767	3,145,791,027	92,748,083

Supplementary Table 1. Assembly statistics. Shown are N50s, the number of contigs, the length of the assembly (bp) as well as the size of the largest contig for all our 14 haplotype-resolved assemblies.

type	variants (unfiltered)	variants (callable regions)	variants (mendelian consistent)	bubbles in pangenome graph
SNP	13,628,117	12,560,841	12,095,177	11,556,580
small insertion	2,229,474	2,163,433	1,922,163	810,298
small deletion	2,026,998	1,961,042	1,811,123	819,445
small complex	0	0	0	597,044
midsize insertion	123,304	120,505	110,882	20,300
midsize deletion	87,263	85,114	80,027	12,720
midsize complex	0	0	0	87,392
large insertion	135,150	123,990	108,929	18,325
large deletion	48,724	45,419	41,499	4,397
large complex	0	0	0	52,272

Supplementary Table 2. **Variant calling statistics.** Numbers of variants obtained at different stages of variant calling/pangenome graph construction. The first column corresponds to the number of raw variant calls made across all individual haplotypes. The second column contains the number of variants within the callable regions, that is, after removing sites with more than 20% of missing ("./.") genotypes. The third column shows to the number of variants left after removing sites with mendelian inconsistencies and corresponds to our final variant callset. The last column presents the number of bubbles in the graph after constructing a pangenome from all variants in the previous column. Columns 1-3 contain only variant alleles that can be classified as SNPs, insertions and deletions. In the graph however, overlapping variant alleles are combined into multi-allelic bubbles. All such bubbles with more than two branches are defined as "complex".

		SNP	small INS	small DEL	midsize INS	midsize DEL	large INS	large DEL
HG00512	total	3,724,332	444,561	442,280	15,635	14,685	14,742	8,623
	unique	251,925	48,811	42,229	5,531	2,831	5,830	1,412
HG00513	total	3,767,798	447,734	444,429	16,025	15,019	15,098	8,900
	unique	258,979	46,955	39,971	5,758	2,978	6,191	1,540
HG00731	total	3,792,925	446,416	448,667	15,630	15,002	15,006	8,717
	unique	237,125	43,607	38,016	5,299	2,585	5,645	1,336
HG00732	total	3,850,476	552,952	469,341	16,943	15,401	15,882	9,082
	unique	258,515	122,268	57,321	6,157	2,961	6,554	1,552
HG02818	total	4,604,971	559,626	566,604	20,166	18,904	17,837	10,740
	unique	605,439	100,220	97,157	8,881	5,455	8,820	2,710
HG03125	total	4,631,416	576,887	580,655	20,290	19,106	18,132	10,759
	unique	608,299	113,155	108,740	9,021	5,574	9,074	2,726
HG03486	total	4,679,604	582,370	575,677	20,421	19,430	18,376	11,027
	unique	670,228	118,933	106,370	9,146	5,935	9,364	2,891
NA12878	total	3,775,211	445,739	448,293	16,029	15,027	15,374	8,777
	unique	247,345	46,445	40,769	5,739	2,816	6,001	1,444
NA19238	total	4,629,589	562,928	584,771	20,099	19,081	18,321	10,870
	unique	606,621	102,902	108,613	8,803	5,496	9,066	2,771
NA19239	total	4,573,111	551,810	575,465	19,611	18,642	17,721	10,664
	unique	589,863	98,383	106,349	8,327	5,466	8,636	2,620
NA24385	total	3,761,904	459,907	447,880	16,144	15,046	15,023	8,769
	unique	247,111	56,090	43,103	5,710	2,792	5,921	1,370
total	total	12,095,177	1,922,163	1,811,123	110,882	80,027	108,929	41,499

Supplementary Table 3. Variants in pangenome graph. Total number of variants detected across all assembly samples ("total"), as well as the number of variants unique to a sample, that is, variants seen only in the respective sample and in none of the other samples ("unique").

		non-repetitive regions	repeat regions	repeat regions [%]
SNPs	biallelic	10,736,632	527,498	4.7 %
	complex	179,476	368,385	67.2 %
small	biallelic INS	682,987	115,702	14.5%
	biallelic DEL	696,243	123,055	15.0 %
	complex INS+DEL	1,238,489	817,458	39.7 %
midsize	biallelic INS	9,313	10,997	54.2 %
	biallelic DEL	5,651	7,909	58.3 %
	complex INS + DEL	43,105	104,734	70.8 %
large	biallelic INS	7,537	10,757	58.8 %
	biallelic DEL	2,212	2,397	52.0 %
	complex INS + DEL	29,277	89,297	75.3 %

Supplementary Table 4. Repetitive regions. Shown are the numbers of variants located inside and outside of STR/VNTR regions for sample NA12878. “biallelic” corresponds to all genomic regions outside of complex bubbles (= bubbles with more than two branches) in our pangenome graph. “complex” corresponds to all callset variants that are located inside of complex bubbles.

coverage	method	NA12878				NA24385			
		time total	time genotyping	memory total	memory genotyping	time total	time genotyping	memory total	memory genotyping
5	PanGenie	21:06:10	19:42:05	84.8	36.4	31:44:24	29:30:54	84.6	36.2
	BayesTyper	27:23:15	26:22:21	39.3	39.3	36:31:30	35:20:37	39.2	39.2
	Platypus	18:12:42	1:20:10	18.2	0.2	20:39:51	1:31:42	8.7	0.1
	GATK[1]	34:41:06	17:24:26	18.2	0.4	35:24:17	15:53:15	8.7	0.4
	Paragraph[2]	39:49:37	22:57:04	18.2	10.1	40:51:58	21:43:48	11.1	11.1
	GraphTyper[3]	22:06:44	5:14:12	18.2	0.2	23:12:02	4:03:52	8.7	0.2
10	PanGenie	21:36:59	19:27:31	84.8	36.4	33:07:31	29:29:26	84.7	36.2
	BayesTyper	38:42:03	37:20:08	40.7	40.7	36:05:15	34:16:52	40.7	40.7
	Platypus	35:20:29	1:42:35	18.6	0.4	42:57:08	1:57:21	8.8	0.3
	GATK[1]	59:42:39	25:21:58	18.6	0.4	67:21:06	25:36:00	8.8	0.5
	Paragraph[2]	66:02:14	32:24:20	18.6	13.2	86:19:41	45:19:54	12.2	12.2
	GraphTyper[3]	42:52:25	9:14:31	18.6	0.3	49:30:28	8:30:41	8.8	0.2
20	PanGenie	23:46:08	19:39:33	84.8	36.4	24:24:09	19:41:24	84.7	36.3
	BayesTyper	32:03:53	29:59:40	41.0	41.0	44:49:37	41:59:38	41.1	41.1
	Platypus	68:38:45	2:11:46	28.4	0.7	81:28:44	2:42:48	8.8	0.5
	GATK[1]	107:04:36	39:18:12	28.4	0.5	120:51:45	40:43:18	8.8	0.8
	ParaGraph[2]	137:18:30	70:51:31	28.4	14.3	139:56:03	61:10:07	12.9	12.9
	GraphTyper[3]	84:34:29	18:07:30	28.4	0.5	92:58:00	14:12:04	8.8	0.3
30	PanGenie	24:58:54	19:31:51	84.8	36.4	26:48:22	19:41:23	84.7	36.3
	Bayestyper	32:24:13	29:34:54	41.1	41.1	48:30:38	44:34:30	44.4	44.4
	Platypus	99:12:01	1:59:29	39.1	1.0	123:09:20	3:02:53	8.8	0.9
	GATK[1]	143:57:46	44:54:12	39.1	0.5	176:26:20	54:21:41	8.8	0.9
	Paragraph[2]	210:28:50	113:16:17	39.1	14.7	256:00:10	135:53:43	13.3	13.3
	GraphTyper[3]	123:03:06	25:50:33	39.1	0.7	141:57:38	21:51:11	8.8	0.5
	Giraffe[3]	3043:47:18	11:10:38	188.7	45.2				

[1] GATK was run on SNPs, small and midsize variants only.
[2] Paragraph was run on midsize and large variants only.
[3] GraphTyper and Giraffe were run on large variants only.

Supplementary Table 5. Resources. Runtime (in CPU hhh:mm:ss) and peak memory usage (in GB) of the different genotyping methods at different coverages. For all methods, we show the total resources needed for producing genotypes from raw, unaligned sequencing reads (“total”), as well as the resources needed only for the genotyping step (“genotyping”). Thus, for Platypus, GATK, Paragraph and GraphTyper the latter excludes the time needed to generate alignments against the reference genome. For Giraffe, it excludes the time for graph construction with vg, indexing and alignment. For k-mer based k-mer based approaches (PanGenie and BayesTyper), it excludes the k-mer counting step. All tools were run on a HPC-cluster predominantly consisting of Intel E5-2697v2 (2× 12 cores and 128 GB of RAM) and Intel Xeon Gold 6136 (2× 12 cores and 192 GB of RAM) nodes.

Table is provided as a separate xlsx file.

Supplementary Table 6. Evaluation of HLA region in haplotype-resolved assemblies. HLA*ASM was used to determine HLA types from our haplotype-resolved assemblies. Table is provided in separate xlsx-file.

	SNP	small INS	small DEL	midsize INS	midsize DEL	large INS	large DEL
unfiltered	12,095,177	1,922,163	1,811,123	110,882	80,027	108,929	41,499
strict	11,234,462	1,198,663	1,202,791	57,699	40,752	56,290	20,490

Supplementary Table 7. Number of variants in strict set. Number of variants contained in the strictly filtered set of all variant types.

	large INS	large DEL
unfiltered	108,929	41,499
lenient	84,836	34,290
strict	56,290	20,490

Supplementary Table 8. Number of variants in filtered and unfiltered sets. Number of variants contained in the unfiltered, strict and lenient set constructed for large variants ($\geq 50bp$).

Table is provided as separate xlsx file.

Supplementary Table 9. LD hits. We show all SVs that were reported in strong linkage disequilibrium with GWAS SNPs ($r^2 >= 0.9$). Coordinates are shown relative to reference genome GRCh38. We show only the most common phenotypes for each GWAS variant[17]. The column “mapped gene(s)" lists the genes mapped to the GWAS SNPs as reported by[17]. If a SNP is located outside of a gene region, the closest upstream and downstream genes are listed separated by a hyphen[17].
